# Quality of outpatient nursing care: a scoping review *

**DOI:** 10.1590/1518-8345.7028.4524

**Published:** 2025-03-31

**Authors:** Bruna Noschang de Brum, Carlise Rigon Dalla Nora, Adriana Roese Ramos, Luciana Foppa, Deise Lisboa Riquinho

**Affiliations:** 1 Universidade Federal do Rio Grande do Sul, Escola de Enfermagem e de Saúde Coletiva, Porto Alegre, RS, Brazil.; 2 Scholarship holder at the Coordenação de Aperfeiçoamento de Pessoal de Nível Superior (CAPES), Brazil.; 3 Hospital de Clínicas de Porto Alegre, Serviço de Enfermagem Ambulatorial, Porto Alegre, RS, Brazil.

**Keywords:** Ambulatory Care, Secondary Care, Nursing, Nursing Care, Health Care Quality Indicators, Quality of Health Care

## Abstract

to map national and international scientific literature on the quality of outpatient nursing care.

a scoping review guided by the Joanna Briggs Institute Collaboration, conducted across 12 databases and repositories. Only original articles evaluating the quality of outpatient nursing care were included. No restrictions were applied regarding time, methodology, or language. Data were mapped and organized through thematic and statistical descriptions of the articles. This review was registered on the Open Science Framework platform.

a total of 45 studies published between 1984 and 2021 were identified, resulting in 17 quality indicators for outpatient nursing care, grouped according to Donabedian’s triad: four for structure, seven for process, and six for outcomes. The predominant area of care was oncology. Among the analyzed studies, 55.3% focused on patients. The most frequently cited indicators were continuing health education, service organization, communication, care coordination, and the nursing process.

the limited number of studies in this field, compared to other areas of nursing, highlights the underexploitation of the topic both nationally and internationally. Additionally, the diversity of identified indicators underscores the lack of standardization in these data.

## Highlights

**(1)** Few studies address the evaluation of outpatient nursing care quality. **(2)** Quality indicators identify challenges in healthcare services. **(3)** Publications on outpatient nursing care quality have increased since 2000. **(4)** Most of the evaluated studies focused on outpatient patients. **(5)** The most frequently cited quality indicator in the studies was Continuing Health Education.

## Introduction

Outpatient care refers to healthcare services provided at this level of care, characterized primarily by the absence of patient hospitalization. These care settings are expanding due to changes in healthcare delivery patterns, population aging, and the increased prevalence of chronic diseases^(1-3)^. In this context, efforts are directed toward improving access to healthcare, with the aim of guiding decision-making^(4)^ to adapt it in ways that promote care improvements.

The evaluation of healthcare quality, widely used as a method to categorize indicators, seeks to encompass different perspectives from users, professionals, and resource providers of various types^(5)^. It is based on Donabedian’s triad of dimensions: structure, process, and outcomes. The structure dimension includes the human, physical, material, and financial resources necessary for care delivery^(5)^. The process dimension encompasses activities performed by healthcare professionals and users, such as diagnosis, treatment, and user-team interactions^(5)^. The outcomes dimension, in turn, refers to the final product of care, considering users’ and professionals’ health, satisfaction, and expectations^(5)^. This categorization allows inferences about care outcomes, as well as the processes that precede them or the environment in which they occur^(5)^.

Two studies available in the literature stand out regarding outpatient quality indicators sensitive to nursing^(6-7)^. A literature review highlighted the following indicators in one study: change in symptom severity, strength of the therapeutic alliance, utilization of services, client satisfaction, risk reduction, increase in protective factors, and level of function/functional status^(6)^. In another study, the cited indicators were: medication reconciliation, controlling high blood pressure, depression assessment conducted, pain assessment and follow-up, and hospital re-admissions^(7)^. Both studies emphasize the need for deeper discussions on quality indicators due to the complexity of the dimensions involved, which complicates the determination and application of quality metrics. It is noteworthy that other important dimensions related to outpatient care were not addressed by the proposed indicators. Nevertheless, the use of quality indicators in nursing is relevant as it enables the visualization of nursing care contributions to patient outcomes.

During the searches, only one scoping review related to the topic of outpatient nursing was found^(8)^. Considering these aspects, this study focused on performance evaluation indicators based on Dubois’ framework and did not include research in Spanish and Portuguese^(8)^, making the development of the present review relevant for producing quality indicators that better reflect the Brazilian and Latin American reality.

Given the relevance of nursing in outpatient care and the belief that it is part of nursing’s responsibilities to ensure the development of quality and safe care practices, this review aims to map national and international scientific literature on the quality of outpatient nursing care.

## Method

### Protocol and registration

This is a scoping review guided by the Joanna Briggs Institute (JBI) Collaboration^(9)^. To ensure transparency and quality, this study followed the guidelines outlined in the Preferred Reporting Items for Systematic Reviews and Meta-Analyses extension for Scoping Reviews (PRISMA-ScR)^(10)^. This scoping review followed these steps: defining and aligning the objective and research question; developing and aligning inclusion criteria with the objective and question; describing the approach; planning the evidence search; selecting, extracting, and presenting the evidence; searching for evidence; selecting evidence; extracting evidence; analyzing evidence; presenting results; summarizing evidence in relation to the review’s purpose, and drawing conclusions and implications from the findings. This review was registered on the Open Science Framework (OSF) platform under the Digital Object Identifier (DOI) https://doi.org/10.17605/OSF.IO/6YP7N.

### Eligibility criteria

The inclusion criteria consisted of original articles addressing the evaluation of outpatient nursing care quality, motivated by the greater methodological rigor and robustness associated with this type of study. Thus, studies with quantitative, qualitative, or mixed designs were included. The selected studies involved nurses providing outpatient care in public or private healthcare services, patients attended by nurses in specialized outpatient settings, or studies describing outpatient nursing care activities.

Exclusion criteria included theoretical studies, reviews, methodological studies, case studies, editorials, experience reports, dissertations, and theses. No temporal or language restrictions were applied to include as many studies as possible.

### Information sources

The studies were selected from various repositories and databases: *Literatura Latino-Americana e do Caribe em Ciências da Saúde* (LILACS), *Índice Bibliográfico Espanhol de Ciências da Saúde* (IBECS), Medical Literature Analysis and Retrieval System Online (Medline) e *Base de Dados de Enfermagem* (BDENF) through the *Biblioteca Virtual em Saúde* (BVS); Scientific Electronic Library Online (SciELO); PubMed; Web of Science Core Collection through Clarivate; Embase and Scopus through Elsevier; Cumulative Index to Nursing and Allied Health Literature (Cinahl) through Ebsco. The search for gray literature was conducted using the Google Scholar tool.

### Search in the literature

The search for studies was conducted on October 22, 2021, and updated on April 30, 2022.

Both the research question and the search strategy implemented in this study were developed using the PCC mnemonic, as follows: P: Population – national and international scientific publications on nursing care quality; C: Concept – quality of nursing care; and C: Context – outpatient services. Thus, the guiding question formulated was: “what do national and international scientific publications report about the quality of nursing care in outpatient services?”.

The search strategy was defined considering the Health Sciences Descriptors (DeCS) and Medical Subject Headings (MeSH) only in English. The descriptors selected were the following: “outpatient care”; “nursing care”; “secondary healthcare”; “quality of healthcare,” maintaining the Boolean operator AND, respecting the peculiarities and characteristics of each database, as shown in Figure 1.

**Figure 1 -:** Search strategies for the databases consulted. Rio Grande do Sul, Brazil, 2023

**Databases**	**Search strategy**
LILACS, IBECS, Medline e BDENF (via *Biblioteca Virtual em Saúde* )	*(“Nursing Care”) AND (“Secondary Care”) AND (“Quality of Health Care”)*
	*(“Nursing Care”) AND (“Ambulatory Care”) AND (“Quality of Health Care”)*
CINAHL (via Ebsco)	*(“Nursing Care”) AND (“Secondary Care”) AND (“Quality of Health Care”)*
	*(“Nursing Care”) AND (“Ambulatory Care”) AND (“Quality of Health Care”)*
SciELO	*(Nursing Care) AND (Secondary Care) AND (Quality of Health Care)*
	*(Nursing Care) AND (Ambulatory Care) AND (Quality of Health Care)*
PubMed	*(Nursing Care) AND (Ambulatory Care) AND (Quality of Health Care)*
	*(“Nursing Care”) AND (“Ambulatory Care”)*
Embase (via Elsevier)	*nursing AND care AND ‘secondary health care’ AND ‘health care quality’*
	*nursing care’ AND ‘ambulatory care’ AND ‘health care quality’*
*Web of Science Core Collection* (via *Clarivate* )	*(Nursing Care) AND (Ambulatory Care) AND (Quality of Health Care)*
	*(Nursing Care) AND (Secondary Care) AND (Quality of Health Care)*
	*(Nursing Care) AND (Ambulatory Care)*
	*(“Nursing Care”) AND (“Secondary Care”)*
	*(“Ambulatory Care”) OR (“Secondary Care”) AND (“Nursing Care”)*
Scopus (via Elsevie *r* )	*(“Nursing Care”) AND (“Ambulatory Care”) AND (“quality off health care”)*
	*(“Nursing Care”) AND (“Secondary Care”) AND (“quality off health care”)*
Google Scholar	*“Nursing Care” AND “Ambulatory Care” AND “Quality of Health Care”*

### Selection of the studies

To organize the articles, they were stored in the *Zotero* ^©^ reference manager. Regarding study selection, the search results were independently analyzed by two researchers using Google Forms^©^ and Google Sheets^©^. Discrepancies were resolved by consensus or with the involvement of a third researcher for evaluation. In other words, the researchers compared the search results, verifying differences in findings, always aiming to include the maximum number of studies possible.

### Data extraction and analysis

For the data extraction phase, a structured form on the Google Forms^©^ platform was used to identify and describe the following items: author, year of publication, country, journal, participants, approach taken, and main results related to the quality of outpatient nursing care, categorizing them according to the dimensions of structure, process, and outcomes.

### Summary of the outcomes

Data were collected from the results of the 45 selected studies, highlighting each study’s relationship with care quality. This data extraction allowed for mapping, interpreting, and performing basic numerical analysis of the scope, nature, and distribution of the studies included in the review. Subsequently, thematic grouping and statistical description of the results were performed using Google Sheets^©^ to provide an overview of all the material. After organizing the results through thematic descriptions, they were grouped into categories based on Donabedian’s triad of structure, process, and outcomes^(5)^, using tables according to their relevance.

The data collection and study selection process will be presented in this article’s results section through a flowchart following PRISMA-ScR guidelines^(10)^.

## Results

A total of 1,530 studies were identified from databases and repositories. After identifying and excluding duplicates, 794 studies remained. These were evaluated by reading titles and abstracts, excluding those not aligned with the theme, resulting in 225 studies for full-text evaluation. After full-text reading, 180 studies were excluded: 119 did not align with the review theme, 48 did not meet inclusion criteria, and 13 could not be retrieved.

**Figure 2 -:**
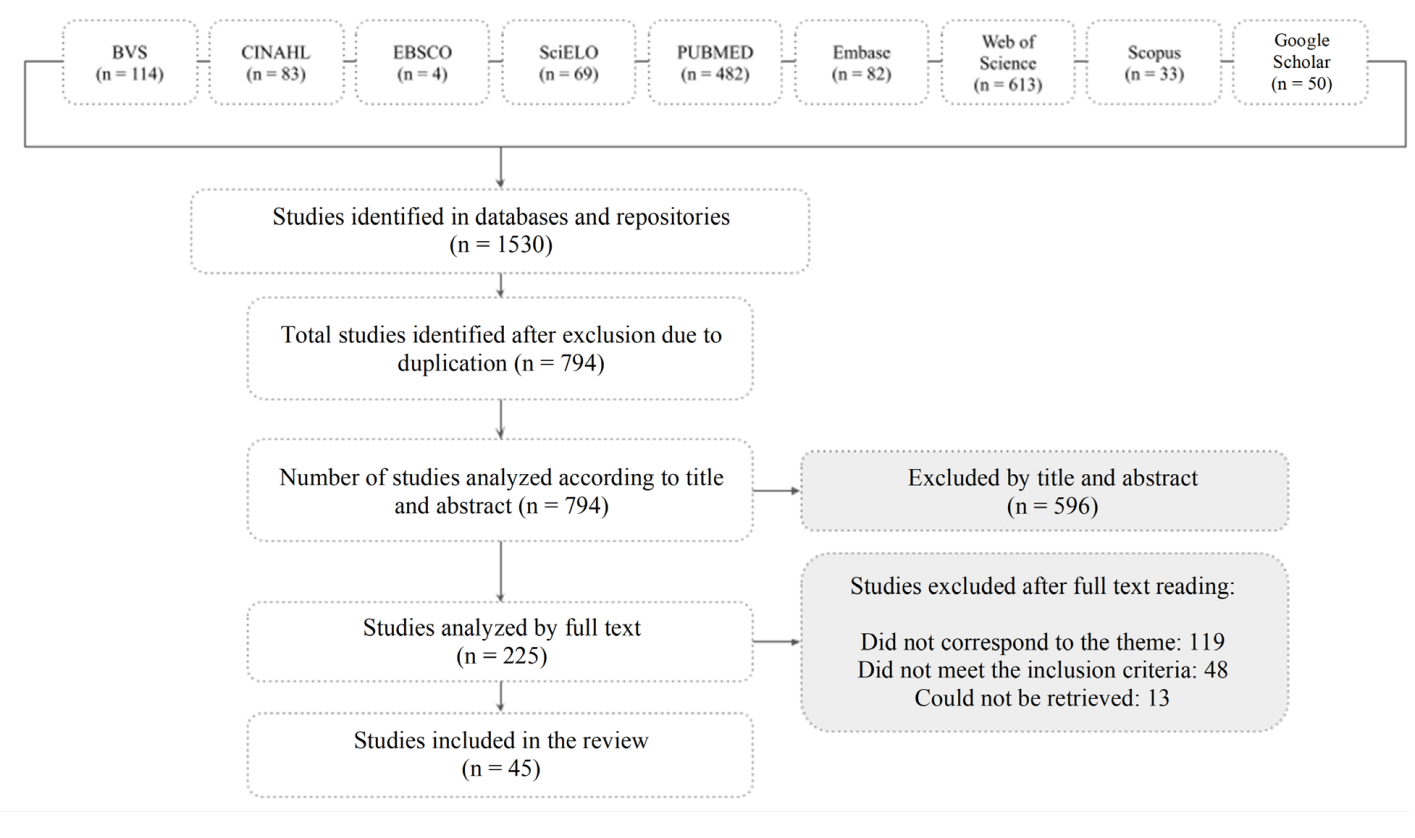
Flowchart of study selection adapted from PRISMA-ScR^(10)^. Rio Grande do Sul, Brazil, 2023

Studies were pre-selected based on title and abstract reading, and the final sample was determined through full-text reading, as shown in the flowchart in Figure 2.

From the selection of articles resulting from database and repository searches, the sample for this review consisted of 45 articles published between 1984 and 2021. The expansion of knowledge in this field can be observed through the increase in publications from 2000 onward, particularly in 2012 and 2018, which showed a higher number of studies compared to other years.

To facilitate presentation, the most relevant data from each article were organized into a table, as shown in Figure 3.

From the analysis of Figure 3, it is concluded that 93.3% of the articles were published after the year 2000, 80% of the articles are in English^(13-29,31,33-44,47-49,51-52,55)^, and 66.7% employed a quantitative methodological approach^(11-12,14-18,21-22,27,29-35,37,40-55)^. Of the 19 identified locations, the United States^(17,19,22,25-26,29,37,39-40,44,47-48,51-52,55)^ was the country with the most published articles (33.3%), followed by Brazil with 12.8%^(11,15,20,24,36,45,53-54)^ and Germany with 8.9%^(12,32,46,50)^.

Regarding the study samples, 55.3% focused on patients^(11,14,18,20-23,25,29,31,35,38,41-49,51-52,54-55)^, followed by 23.4% focusing on nurses^(17,24,26-28,30,34,36-37,39-40)^, 10.6% on nursing records^(13,15-16,33,50)^, 4.3% on family members of patients^(19,53)^, 4.3% on healthcare professionals^(19,32)^, and 2.1% on quality indicators^(12)^. The predominant care area among the articles was oncology^(21,27-31,33,39-42,45,47-48)^, accounting for 31.1% of the publications.

From the thematic grouping of the articles in this review, considering the research question and proposed objective, 17 indicators influencing the quality of nursing care were identified and are presented in Figure 4. The distribution of these indicators was as follows: 43.2% in the process dimension^(12-23,25-28,30-35,37-39,41-54)^, 30.3% in the outcome dimension^(11,18,20-24,28-29,31-32,35-47,49,51-55)^, and 26.5% in the structure dimension^(13-14,16-22,24,27,34-37,39-41,43-49,51-52,55)^.

**Figure 3 -:** Characterization of articles by author, year of publication, country of study, language, approach, sample, and care area. Rio Grande do Sul, Brazil, 2023

**Citation**	**Year/Country/Language**	**Approach**	**Sample**	**Care Area**
Cunha, et al., 2021 ^(11)^	2021	Quantitative	Patients	Cardiology
	Brazil	(longitudinal)		
	Portuguese			
Seibert, et al., 2020 ^(12)^	2020	Quantitative	Quality indicators	Geriatrics
	Germany	(cross-sectional)		
	German			
Ameel, et al., 2020 ^(13)^	2020	Qualitative	Nursing records*	Psychiatry
	Finland	(descriptive)		
	English			
Zendrato; Hariyati; Afifah, 2019 ^(14)^	2019	Quantitative	Patients	Polyclinic
	Indonesia	(descriptive)		
	English			
Errico, et al., 2018 ^(15)^	2018	Quantitative	Nursing records*	Obstetrics
	Brazil	(cross-sectional)		
	English			
Heale, et al., 2018 ^(16)^	2018	Quantitative	Nursing records*	Endocrinology
	Canada	(cross-sectional)		
	English			
Connor, et al., 2018 ^(17)^	2018	Quantitative	Nurses ^†^	Pediatric cardiology
	USA	(descriptive)		
	English			
Seabra; Amendoeira; Sá, 2018 ^(18)^	2018	Quantitative	Patients	Psychiatry
	Portugal	(cross-sectional)		
	English			
Anderson, et al., 2018 ^(19)^	2018	Qualitative	Health professionals	Pediatric cardiology
	USA	(descriptive)	Family members of patients	
	English			
Silva, et al., 2018 ^(20)^	2018	Qualitative	Patients	Stomatherapy
	Brazil	(descriptive)		
	English			
Calvo; Sepulveda-Carrillo, 2017 ^(21)^	2017	Quantitative	Patients	Oncology
	Colombia	(cross-sectional)		
	English			
Ye, et al., 2016 ^(22)^	2016	Quantitative	Patients	Pediatrics
	USA	(longitudinal)		
	English			
Selvin, et al., 2016 ^(23)^	2016	Qualitative	Patients	Psychiatry
	Switzerland	(descriptive)		
	English			
Macedo, et al., 2016 ^(24)^	2016	Qualitative	Nurses ^†^	HIV/AIDS Outpatient Care
	Brazil	(descriptive)		
	English			
Vessey, et al., 2015 ^(25)^	2015	Qualitative	Patients	Pediatrics
	USA	(descriptive)		
	English			
Vanderboom; Thackeray; Rhudy, 2015 ^(26)^	2015	Qualitative	Nurses ^†^	Polyclinic
	USA	(descriptive)		
	English			
Tuna, et al., 2015 ^(27)^	2015	Quantitative	Nurses ^†^	Oncology
	Turkey	(descriptive)		
	English			
Komatsu; Yagasaki, 2014 ^(28)^	2014	Qualitative	Nurses ^†^	Oncology
	Japan	(cross-sectional)		
	English			
Hammelef, et al., 2014 ^(29)^	2014	Quantitative	Patients	Oncology
	USA	(cross-sectional)		
	English			
Font Difour, et al., 2014 ^(30)^	2014	Quantitative	Patients and	Oncology
	Cuba	(descriptive)	Nurses ^†^	
	Spanish			
Armes, et al., 2014 ^(31)^	2014	Quantitative	Patients	Oncology
	England	(cross-sectional)		
	English			
Van Den Bussche, et al., 2013 ^(32)^	2013	Quantitative	Health professionals	Psychiatry
	Germany	(descriptive)		
	German			
Palese, et al., 2013 ^(33)^	2013	Quantitative	Nursing records*	Oncology
	Italy	(cross-sectional)		
	English			
Callen, et al., 2013 ^(34)^	2013	Mixed	Nurses ^†^	Rheumatology
	Australia			
	English			
Williams, et al., 2012 ^(35)^	2012	Quantitative	Patients	Cardiology
	United Kingdom	(descriptive)		
	English			
Pinto, et al., 2012 ^(36)^	2012	Qualitative	Nurses ^†^	No Description
	Brazil	(descriptive)		
	English			
Pfeiffer, et al., 2012 ^(37)^	2012	Mixed	Nurses ^†^	No Description
	USA			
	English			
Larsson, et al., 2012 ^(38)^	2012	Qualitative	Patients	Rheumatology
	Sweden	(descriptive)		
	English			
Kamimura, et al., 2012 ^(39)^	2012	Qualitative	Nurses ^†^	Oncology
	USA	(descriptive)		
	English			
Friese; Manojlovich, 2012 ^(40)^	2012	Mixed	Nurses ^†^	Oncology
	USA			
	English			
Hjoerleifsdottir; Hallberg; Gunnarsdottir, 2010 ^(41)^	2010	Quantitative	Patients	Oncology
	Iceland	(cross-sectional)		
	English			
Skrutkowski, et al., 2008 ^(42)^	2008	Quantitative	Patients	Oncology
	Canada	(interventional)		
	English			
Rootmensen, et al., 2008 ^(43)^	2008	Quantitative	Patients	Pneumology
	Netherlands	(interventional)		
	English			
Sisk, et al., 2006 ^(44)^	2006	Quantitative	Patients	Cardiology
	USA	(interventional)		
	English			
Fonseca; Gutiérrez; Adami, 2006 ^(45)^	2006	Quantitative	Patients	Oncology
	Brazil	(descriptive)		
	Portuguese			
Mohrmann, et al., 2005 ^(46)^	2005	Quantitative	Patients	No Description
	Germany	(descriptive)		
	German			
Gesell; Gregory, 2004 ^(47)^	2004	Quantitative	Patients	Oncology
	USA	(descriptive)		
	English			
Cusack; Jones-Wells; Chisholm, 2004 ^(48)^	2004	Quantitative	Patients	Oncology
	USA	(descriptive)		
	English			
Arthur; Clifford, 2004 ^(49)^	2004	Quantitative	Patients	Rheumatology
	United Kingdom	(cross-sectional)		
	English			
Zink, et al., 2000 ^(50)^	2000	Quantitative	Nursing records*	No Description
	Germany	(descriptive)		
	German			
Oermann; Templin, 2000 ^(51)^	2000	Quantitative	Patients	No Description
	USA	(cross-sectional)		
	English			
Oermann; Dillon; Templin, 2000 ^(52)^	2000	Quantitative	Patients	No Description
	USA	(descriptive)		
	English			
Sanna, 1993 ^(53)^	1993	Qualitative	Family members of patients	Pediatrics
	Brazil	(descriptive)		
	Portuguese			
Silva, 1985 ^(54)^	1985	Quantitative	Patients	Endocrinology
	Brazil	(interventional)		
	Portuguese			
Chang, et al., 1984 ^(55)^	1984	Quantitative	Patients	Geriatrics
	USA	(interventional)		
	English			

*Nursing Records = Includes all types of documentation completed by the nursing team; ^†^Nurses = Due to the diversity of study locations and different nursing organization structures, the population was standardized as nurses, but it includes various professionals within this category

**Figure 4 -:** Quality indicators for outpatient nursing care according to Donabedian’s triad of structure, process, and outcomes^(5)^. Rio Grande do Sul, Brazil, 2023

**Structure**	**Process**	**Results**
Staffing ^(27 , 36 , 39 - 40 , 45)^	Nursing competencies ^(30 - 31 , 34 - 35 , 37 , 40 , 46)^	Self-Care ^(11 , 20 - 21 , 23 , 31 , 42 - 44 , 52 , 54)^
Continuing Health Education ^(14 , 16 , 19 , 24 , 36 - 37 , 43 , 45 - 46 , 48 - 49 , 51 - 52 , 55)^	Communication ^(14 , 16 , 22 - 23 , 32 , 37 - 41 , 51 - 52)^	Nurse-Team Relationship ^(24 , 32 , 37 , 39 - 40)^
Infrastructure ^(14 , 16 , 22 , 36 , 39 - 41 , 45 , 47)^	Care coordination ^(15 - 17 , 25 - 26 , 28 , 32 , 40 , 42 , 44 , 47 , 49)^	Nurse-Patient Relationship ^(22 - 23 , 28 , 38 , 45 , 47 , 49 , 51 - 53 , 55)^
Service organization ^(13 , 16 , 20 , 22 , 34 - 36 , 39 , 41 , 44 , 46 - 47 , 52)^	Nursing care ^(13 , 16 , 18 , 20 , 27 , 41 , 43 , 46 , 48 , 53)^	Patient satisfaction ^(18 , 35 , 38 , 41 , 45 - 46 , 49 , 51 - 53 , 55)^
	Diagnosis ^(12 , 15 , 18)^	Professional satisfaction ^(36 - 37 , 39)^
	Health education ^(17 , 21 , 41 - 45 , 47 , 51 - 52 , 54)^	Mental health ^(18 , 21 , 28 - 29 , 35 , 42 , 47)^
	Nursing process ^(13 - 16 , 19 , 26 , 30 , 31 , 46 - 48 , 50)^	

### Quality indicators in the structure dimension

The studies included in this category identified four indicators: staffing, continuing health education, infrastructure, and service organization.

Staffing^(27,36,39-40,45)^ involves human resources, workload, team sizing, the number of patients per professional based on each patient’s care needs and the required level of expertise, as well as the availability and accessibility of healthcare professionals.

Continuing health education^(14,16,19,24,36-37,43,45-46,48-49,51-52,55)^ refers to educational activities aimed at healthcare professionals, including technical skills, theoretical and practical knowledge, professional experience, ongoing training, feedback, and evidence-based practices.

Infrastructure^(14,16,22,36,39-41,45,47)^ relates to the physical facilities of healthcare services, the availability of material and community resources, waiting times for care, and accessibility to healthcare services.

Service organization^(13,16,20,22,34-36,39,41,44,46-47,52)^ encompasses organizational aspects of healthcare entities, such as access, staffing, the prevailing care model, managerial activities, auditing processes, and health information technologies.

### Quality indicators in the process dimension

The studies included in this category identified seven indicators: nursing competencies, communication, care coordination, nursing care, diagnosis, health education, and the nursing process.

Nursing competencies^(30-31,34-35,37,40,46)^ include activities related to the skills required of nurses, such as leadership, team and material resource management, teamwork, conflict resolution, administrative tasks, patient safety, supervision, and care management.

Communication^(14,16,22-23,32,37-41,51-52)^ involves the nurse’s ability to convey knowledge to patients, families, and the community, ensuring that instructions are understood by patients and that professionals comprehend their needs through effective information exchange. This requires attention, empathy, sensitivity, assertiveness, and respect to facilitate effective communication.

Care coordination^(15-17,25-26,28,32,40,42,44,47,49)^ refers to activities carried out in collaboration with other healthcare professionals, such as referrals, continuity of care based on patient needs, scheduling appointments and exams, prescription renewals, and patient and family follow-up.

Nursing care^(13,16,18,20,27,41,43,46,48,53)^ involves direct patient care activities such as nursing interventions, assessing care intensity, team and patient characteristics, triage, and comprehensive care. Diagnosis^(12,15,18)^ pertains to the pathologies presented by patients according to the International Classification of Diseases (ICD).

Health education^(17,21,41-45,47,51-52,54)^ includes educational activities aimed at guiding patients and the community about health issues and providing information for health promotion, prevention, and recovery. Articles highlight counseling on healthy habits, disease and procedure information, adverse event prevention, and reducing material resource waste.

The nursing process^(13-16,19,26,30,31,46-48,50)^ refers to all activities encompassing the five stages of the nursing process: data collection, diagnosis, planning, implementation, and evaluation, including nursing documentation.

### Quality indicators in the outcome dimension

The studies included in this category identified six indicators: self-care, nurse-team relationship, nurse-patient relationship, patient satisfaction, professional satisfaction, and mental health.

Self-care^(11,20-21,23,31,42-44,52,54)^ relates to patient participation in the care process and symptom management. The initiative of the patient and their family members to promote and maintain health, adhere to treatment, and develop activities that encourage the patient to become an active agent and co-responsible for their own care are part of the strategies mentioned in the studies.

The nurse-team relationship^(24,32,37,39-40)^ refers to the nurse’s interaction with colleagues, whether nursing or other professionals. It involves respect, understanding, trust, communication, availability, behavior, autonomy, and conflict management.

The nurse-patient relationship^(22-23,28,38,45,47,49,51-53,55)^ pertains to the behavioral dynamics between nurses and patients. Encouraging autonomy, patient follow-up, cordiality-based encounters, empathy, active listening, understanding feelings, acceptance, and sensitivity foster trust and security.

Patient satisfaction^(18,35,38,41,45-46,49,51-53,55)^ is related to the patient’s perception of care outcomes. Satisfaction levels are influenced by care quality, values alignment, participation in care decisions, health-related information provided, professional-patient rapport, and the care environment.

Professional satisfaction^(36-37,39)^ refers to healthcare professionals’ perceptions of their work activities. Satisfaction or dissatisfaction levels are influenced by job responsibilities, team dynamics, and healthcare facility conditions.

Mental health^(18,21,28-29,35,42,47)^ encompasses the emotional needs of both patients and professionals. Psychological distress can be influenced by conflicts and trust levels between healthcare professionals and patients or among team members. The development of educational activities on mental well-being and referrals for emotional support based on individual needs can mitigate these issues.

## Discussion

The findings of this review assist in mapping knowledge production in specialized outpatient nursing. A significant portion of the studies reflects the international context of nursing practice, which may limit their applicability to the Brazilian reality. Most articles focused on patients attended by nursing teams, highlighting the patient’s importance in developing service quality levels^(11,14,18,20-23,25,29,31,35,38,41-49,51-52,54-55)^. Although certain nursing specialties involved in outpatient care have a higher volume of studies, such as oncology^(21,27-31,33,39-42,45,47-48)^, the studies were conducted in conjunction with hospital settings^(56)^, making it difficult to determine whether the results accurately reflect the outpatient reality.

Nursing plays a critical role in outpatient care by implementing patient- and family-centered practices. Quality indicators should reflect the nature of interventions and their concerns^(8)^. In our analysis, we identified four structure-related health indicators—staffing, continuing health education, infrastructure, and service organization; seven process-related indicators—nursing competencies, communication, care coordination, nursing care, diagnosis, health education, and the nursing process; and six outcome-related indicators—self-care, nurse-team relationship, nurse-patient relationship, patient satisfaction, professional satisfaction, and mental health. The literature has indicated a greater focus on using process indicators to improve service quality, as these are strongly associated with outcomes^(56)^. Improving indicators in these two dimensions can generate significant impacts on healthcare services^(56)^.

The connection between nursing care and patient well-being is complex. Inadequate support for the nursing workforce can profoundly affect care quality, negatively impacting patient health^(8)^. Excessive workloads, lack of organizational structure in healthcare services, and staff turnover compromise community relationships and influence care quality^(56-57)^. Additionally, frequent staff changes overload teams due to the constant need to train new members, increasing costs and weakening workflows^(57)^. A lower patient-to-nurse ratio has been associated with better health outcomes^(8)^, as settings where patient-to-nurse ratios are based on nursing care provide higher-quality, evidence-based practices^(8)^. In California, the National Nurses United employs a fixed nurse-to-patient ratio model, establishing mandatory staffing levels based on the environment and patients’ health conditions^(58)^.

Nurses’ management roles are often reduced to bureaucratic and organizational tasks, overburdening professionals who must balance care production with administrative duties such as personnel and supply management^(57)^. Despite the importance of healthcare service organization in ensuring continuity of patient care^(59-60)^, the bureaucratization and mechanization of nursing work jeopardize patient health^(57)^, potentially leading to the delegation of direct care to nursing technicians and assistants^(57)^.

Some health conditions may require specialized treatment and coordination between primary and secondary care levels. Coordination between these levels can be challenging; a lack of trust and knowledge among professionals may result in unnecessary referrals to specialized services. However, these services are often under-resourced and overburdened^(61)^. Referrals are a critical component of patient management, ensuring continuity of care^(62)^. Effective matrix support provided by specialized services would enhance the technical capacity and confidence of professionals to handle such cases, improving the resolution of primary care and avoiding unnecessary referrals^(61)^.

Some studies report that difficulties accessing healthcare services are a primary reason for missed appointments, which hinders follow-up and continuity of care^(60)^. It is suggested that as continuity percentages increase, health outcomes improve^(56)^, as continuity fosters trust between professionals and patients, facilitating effective communication and improving treatment adherence^(56)^.

Studies addressing communication formed part of our sample. Patients identify communication as a high-quality healthcare indicator; effective communication between professionals and patients translates into better outcomes^(63)^. Attributes of patient communication should include listening skills, respect, courtesy, clear explanations, and appropriate language^(63)^. Furthermore, effective communication should also occur among professionals and between healthcare institutions^(63)^.

Some studies discuss nursing records, which include all documentation completed by nurses and are essential for ensuring care quality and patient safety^(59)^. The documentation of the steps in the nursing process—anamnesis and physical examination, nursing diagnosis, nursing prescription, and evaluation^(64)^—is an important tool for continuity of care, serving as a record of the activities performed by the team, ensuring the provision of key information about care, and preserving relevant data for the auditing process^(59)^. However, nursing records have been identified as one of the areas with the greatest quality deficits in the nursing process in Brazil. Despite their recognized importance, barriers such as staff shortages, excessive workloads, and lack of theoretical knowledge hinder proper recordkeeping^(59)^.

Among other essential nursing competencies are technical-scientific knowledge, relational skills, and administrative, care-related, and personnel management abilities. Maintaining appropriate relationships with patients, families, and team members requires commitment, involvement, and ethical conduct^(57)^. Nursing leadership is indispensable for creating positive work environments and is strongly linked to professional satisfaction and improved mental health. However, its use is not limited to the management of healthcare teams; it is also highly relevant for promoting preventive care.

Recognizing that patients themselves are the most qualified to provide information about what is important in care and interactions with healthcare professionals^(65)^ has a significant impact on patient quality and satisfaction, as evidence points to an association between positive experiences, better outcomes, and greater adherence to treatment^(65)^. Patient experience data, typically obtained through satisfaction surveys, can be used as performance information at all levels of the healthcare system, as they provide robust data on care delivery^(65-66)^.

The health education indicator influences patient outcomes and is a key component of nursing practice^(56)^, as it enhances the patient’s self-management capacity, reducing the risk of acute health conditions in both the short and long term^(56)^. Although the importance of health education for care quality is well-documented in the literature, few articles detail the educational activities implemented or evaluate the impact of these interventions on patients.

Education is also a critical factor in training healthcare professionals. The Pan American Health Organization (PAHO) has already warned about the training of healthcare professionals under a university curriculum based on curative paradigms, hospital-centered approaches, and fragmented health knowledge, prioritizing specialties while neglecting a holistic understanding of human beings and the health-disease processes^(57)^. For this reason, continuing education is an important tool for improving nursing work and should consider each context and workplace demands^(57)^.

Evidence-based practice was present in the analyzed studies. This method is defined as an approach to solving health problems and improving decision-making, guided by the search for the best and most recent evidence, which includes clinical experience, patient evaluation, and preferences within a healthcare context. The studies indicated that evidence-based practice enhances healthcare system quality, improves patient outcomes, reduces costs, and promotes greater satisfaction^(62)^. The self-care indicator is strongly embedded in the scope of nursing practice, as nurses routinely provide this service through health education practices^(67)^.

The findings from this review contribute to advancing scientific knowledge in the field of nursing within public health, particularly in improving the quality of outpatient care. The evidence collected helps bridge the knowledge gap regarding outpatient nursing quality indicators, given the significant role nurses play in care management. However, there appear to be barriers to translating research findings into practical care settings. Future research in this area could investigate these barriers, as such indicators have long been widely used in hospital care.

This study faced limitations in obtaining specific research on specialized outpatient nursing. Although the topic of outpatient nursing quality is not new in the literature, research production at this level of care remains limited compared to the large volume of studies conducted in hospital and primary care settings. Additionally, the difficulty in accessing primary articles focused on specialized outpatient care restricted the ability to make in-depth inferences. A larger set of primary data would be necessary to ensure a more comprehensive analysis. As a result, the relationships discussed in this article were primarily derived from studies focusing on other outpatient configurations.

The use of different descriptors or indexing databases not included in this review could potentially yield additional studies. Furthermore, limiting the review to original articles, not using descriptors in other languages—such as Portuguese and Spanish—or relevant synonyms, as well as omitting other descriptors—such as Cinahl’s Thesaurus and Embase’s Emtree—may have introduced bias in study identification. Thus, the authors acknowledge that important published research may have been omitted due to the search strategy employed.

The mapped studies reveal disparities in national and international publications on the topic, with limited exploration in the Brazilian context, as well as a diversity of indicators influencing nursing care quality. Therefore, there appears to be a gap in scientific knowledge regarding the standardization of outpatient nursing quality indicators.

## Conclusion

The mapping of national and international scientific literature on the quality of outpatient nursing care revealed limited exploration of the topic, especially when compared to other areas of outpatient nursing knowledge, both nationally and internationally. From the thematic grouping of the analyzed data, 17 indicators emerged that influence the quality of nursing care, most of which fall under the process dimension. The most frequently cited indicators were: continuing health education, service organization, communication, care coordination, and the nursing process. The diversity of indicators highlights a lack of standardization in these data.

Thus, this study contributes to the development of outpatient nursing quality indicators, aiming to address the need for deeper knowledge production at the outpatient level. Quality indicators are part of a strategy for mapping challenges and investment needs in healthcare service structures. They can be used to guide and support actions and decisions related to care practices, ensuring reliable, safe, and effective nursing care. In the absence of appropriate methods for evaluating indicators, low scores may actually reflect weaknesses and inadequacies in the healthcare provided, such as underreporting, failures in detecting health issues, and inadequate treatments.

Ensuring access to quality healthcare services is essential. To achieve this, different levels of healthcare must work together to avoid overburdening services and deteriorating care quality, which leads to professional burnout, repeated hospital readmissions, and the chronicity of acute conditions.

## References

[1] Borges M. M., Custódio L. A., Cavalcante D. F. B., Pereira A. C., Carregaro R. L. (2023). Direct healthcare cost of hospital admissions for chronic non-communicable diseases sensitive to primary care in the elderly. Cien Saude Colet.

[2] Nehme M., Arsever S., Tahar A., Lidsky D., Chevallier Lugon C., Braillard O. (2024). Nouveautés et perspectives en médecine interne générale ambulatoire. Rev Med Suisse.

[3] Hebert P. L., Kumbier K. E., Smith V. A., Hynes D. M., Govier D. J., Wong E. (2024). Changes in Outpatient Health Care Use After COVID-19 Infection Among Veterans. JAMA Netw Open.

[4] Amado G. C., Ferreira D. C., Nunes A. M. (2022). Vertical integration in healthcare: What does literature say about improvements on quality, access, efficiency, and costs containment?. Int J Health Plann Manage.

[5] Donabedian A. (1988). The quality of care. How can it be assessed?. JAMA.

[6] Sawyer L. M., Berkowitz B., Haber J. E., Larrabee J. H., Marino B. L., Martin K. S. (2002). Expanding American Nurses Association nursing quality indicators to community-based practices. Outcomes Manag [Internet].

[7] Martinez K., Battaglia R., Start R., Mastal M. F., Matlock A. M. (2015). Nursing-Sensitive Indicators in Ambulatory Care. Nurs Econ [Internet].

[8] Rapin J., D’Amour D., Dubois C. A. (2015). Indicators for Evaluating the Performance and Quality of Care of Ambulatory Care Nurses. Nurs Res Pract.

[9] Peters M. D. J., Godfrey C., McInerney P., Munn Z., Tricco A. C., Khalil H., Aromataris E., Munn Z. (2020). JBI Manual for Evidence Synthesis.

[10] Tricco A. C., Lillie E., Zarin W., O’Brien K. K., Colquhoun H., Levac D. (2018). PRISMA extension for scoping reviews (PRISMA-ScR): checklist and explanation. Ann Intern Med.

[11] Cunha D. C. P. T., Rossi L. A., Dessote C. A. M., Bolela F., Dantas R. A. S. (2021). Evolution of self-care in patients with heart failure at the first outpatient return and three months after hospital discharge. Rev. Latino-Am. Enfermagem.

[12] Seibert K., Stiefler S., Domhoff D., Wolf-Ostermann K., Peschke D. (2020). Quality of ambulatory medical care in the context of age and care-dependency: Results of a cross-sectional analysis of German health claims data. Z Evid Fortbild Qual Gesundhwes.

[13] Ameel M., Leino H., Kontio R., Achterberg T., Junttila K. (2020). Using the Nursing Interventions Classification to identify nursing interventions in free-text nursing documentation in adult psychiatric outpatient care setting. J Clin Nurs.

[14] Zendrato M. V., Hariyati R. T. S., Afifah E. (2019). Outpatient nursing care implementations in Indonesian regional public hospitals. Enferm Clin.

[15] Errico L. S. P., Bicalho P. G., Oliveira T. C. F. L., Martins E. F. (2018). The work of nurses in high-risk prenatal care from the perspective of basic human needs. Rev Bras Enferm.

[16] Heale R., Wenghofer E., James S., Garceau M. L. (2018). Quality of Care for Patients With Diabetes and Mulitmorbidity Registered at Nurse Practitioner-Led Clinics. Can J Nurs Res.

[17] Connor J. A., Antonelli R. C., O’Connell C. A., Kuzdeba H. B., Porter C., Hickey P. A. (2018). Measuring Care Coordination in the Pediatric Cardiology Ambulatory Setting. J Nurs Adm.

[18] Seabra P. R. C., Amendoeira J. J. P., Sá L. O. (2018). Testing Nursing Sensitive Outcomes in Out-Patient Drug Addicts, with "Nursing Role Effectiveness Model". Issues Ment Health Nurs.

[19] Anderson J. B., Chowdhury D., Connor J. A., Daniels C. J., Fleishman C. E., Gaies M. (2018). Optimizing patient care and outcomes through the congenital heart center of the 21st century. Congenit Heart Dis.

[20] Silva J. A. A., Rodrigues S. O., Abreu C. S. S., Santos R. R., Pieszak G. M., Durgante V. L. (2018). The therapeutic route of chronic venous ulcer bearing patients and its effects towards nursing care. Rev Fundam Care Online.

[21] Calvo L. E. A., Sepulveda-Carrillo G. J. (2017). Care needs of cancer patients undergoing ambulatory treatment. Enferm Glob.

[22] Ye G., Rosen P., Collins B., Lawless S. (2016). One Size Does Not Fit All: Pediatric Patient Satisfaction Within an Integrated Health Network. Am J Med Qual.

[23] Selvin M., Almqvist K., Kjellin L., Schoder A. (2016). The Concept of Patient Participation in Forensic Psychiatric Care: The Patient Perspective. J Forensic Nurs.

[24] Macedo S. M., Miranda K. C. L., Silveira L. C., Gomes A. M. T. (2016). Nursing care in Specialized HIV/Aids Outpatient Services. Rev Bras Enferm.

[25] Vessey J. A., McCrave J., Curro-Harrington C., DiFazio R. L. (2015). Enhancing Care Coordination Through Patient- and Family-Initiated Telephone Encounters: A Quality Improvement Project. J Pediatr Nurs.

[26] Vanderboom C. E., Thackeray N. L., Rhudy L. M. (2015). Key factors in patient-centered care coordination in ambulatory care: Nurse care coordinators’ perspectives. Appl Nurs Res.

[27] Tuna R., Baykal U., Turkmen E., Yildirim A. (2015). Planning for the Size of the Nursing Staff at an Outpatient Chemotherapy Unit. Clin J Oncol Nurs.

[28] Komatsu H., Yagasaki K. (2014). The Power of nursing: Guiding patients through a journey of uncertainty. Eur J Oncol Nurs.

[29] Hammelef K. J., Friese C. R., Breslin T. M., Riba M., Schneider S. M. (2014). Implementing Distress Management Guidelines in Ambulatory Oncology: A Quality Improvement Project. Clin J Oncol Nurs.

[30] Difour M. V. F., Legra B. E., Tumbarell N. T., Pineda Y. B., Bonne A. H. S. (2014). Evaluation of quality of the nursing care in oncology patients treated with chemotherapy. MEDISAN [Internet].

[31] Armes J., Wagland R., Finnegan-John J., Richardson A., Corner J., Griffiths P. (2014). Development and Testing of the Patient-Reported Chemotherapy Indicators of Symptoms and Experience Patient-Reported Outcome and Process Indicators Sensitive to the Quality of Nursing Care in Ambulatory Chemotherapy Settings. Cancer Nurs.

[32] H Bussche, ÄD Jahncke-Latteck, A Ernst, B Tetzlaff, B Wiese, U Schramm (2013). Satisfied General Practitioners and Critical Nursing Staff - Problems of Interprofessional Cooperation in the Home Care of Dementia Patients. Gesundheitswesen.

[33] Palese A., Zanini A., Carlevaris E., Morandin A., Carpanelli I., Dante A. (2013). Hidden outpatient oncology Clinical Nursing Minimum Data Set: Findings from an Italian multi-method study. Eur J Oncol Nurs.

[34] Callen J., Hordern A., Gibson K., Li L., Hains I. M., Westbrook J. I. (2013). Can technology change the work of nurses? Evaluation of a drug monitoring system for ambulatory chronic disease patients. Int J Med Inform.

[35] Williams S., Williams J., Tcherveniakov P., Milton R. (2012). Impact of a thoracic nurse-led chest drain clinic on patient satisfaction. Interact Cardiovasc Thorac Surg.

[36] Pinto I. C., Marciliano C. S. M., Zacharias F. C. M., Stina A. P. N., Passeri I. A. G., Bulgarelli A. F. (2012). Nursing care practices at an outpatient care center from an integrative perspective. Rev. Latino-Am. Enfermagem.

[37] Pfeiffer J. A., Wickline M. A., Deetz J., Berry E. S. (2012). Assessing RN-to-RN peer review on clinical units. J Nurs Manag.

[38] Larsson I., Bergman S., Fridlund B., Arvidsson B. (2012). Patients’ experiences of a nurse-led rheumatology clinic in Sweden: a qualitative study. Nurs Health Sci.

[39] Kamimura A., Schneider K., Lee C. S., Crawford S. D., Friese C. R. (2012). Practice environments of nurses in ambulatory oncology settings: A thematic analysis. Cancer Nurs.

[40] Friese C. R., Manojlovich M. (2012). Nurse-physician relationships in ambulatory oncology settings. J Nurs Sch.

[41] Hjoerleifsdottir E., Hallberg I. R., Gunnarsdottir E. D. (2010). Satisfaction with care in oncology outpatient clinics: psychometric characteristics of the Icelandic EORTC IN-PATSAT32 version. J Clin Nurs.

[42] Skrutkowski M., Saucier A., Eades M., Swidzinski M., Ritchie J., Marchionni C. (2008). Impact of a Pivot Nurse in Oncology on Patients With Lung or Breast Cancer: Symptom Distress, Fatigue, Quality of Life, and Use of Healthcare Resources. Oncol Nurs Forum.

[43] Rootmensen G. N., Keirapema A. R. J., Looysen E. E., Schaaf L., Haan R. J., Jansen H. M. (2008). The effects of additional care by a pulmonary nurse for asthma and COPD patients at a respiratory outpatient clinic: Results from a double blind, randomized clinical trial. Patient Educ Couns.

[44] Sisk J. E., Hebert P. L., Horowitz C. R., McLaughlin M. A., Wang J. J., Chassin M. R. (2006). Effects of nurse management on the quality of heart failure care in minority communities: a randomized trial. Ann Intern Med.

[45] Fonseca S. M., Gutiérrez M. G. R., Adami N. P. (2006). Evaluation of the satisfaction level of cancer patients with the assistance received during ambulatory antineoplastic chemotherapy. Rev Bras Enferm.

[46] Mohrmann M., Lotz-Metz G., Böhler T., Hannes W. (2005). The nursing process as an instrument for quality assurance of out-patient nursing services. Gesundh Ökon Qual Manag.

[47] Gesell S. B., Gregory N. (2004). Identifying priority actions for improving patient satisfaction with outpatient cancer care. J Nurs Care Qual.

[48] Cusack G., Jones-Wells A., Chisholm L. (2004). Patient intensity in an ambulatory oncology research center: A step forward for the field of ambulatory care. Nurs Econ [Internet].

[49] Arthur V., Clifford C. (2004). Rheumatology: a study of patient satisfaction with follow-up monitoring care. J Clin Nurs.

[50] Zink J., Zenz A., Bokelmann M., Mohrmann M., Schwoerer P. (2000). Quality control of outpatient nurse care services – outcome of an area-wide investigation implemented by the Health Insurance Medical Service (MDK) of Baden-Württemberg. Gesundheitswesen.

[51] Oermann M. H., Templin T. (2000). Important attributes of quality health care: Consumer perspectives. J Nurs Scholarsh.

[52] Oermann M. H., Dillon S. L., Templin T. (2000). Indicators of quality of care in clinics: patients’ perspectives. J Healthc Qual.

[53] Sanna M. C. (1993). The evaluation of outpatient nursing care according to the client’s perception. Rev Esc Enferm USP.

[54] Silva H. M. (1985). Programa de Assistência Ambulatorial de Enfermagem para Clientes Diabéticos. Rev Bras Enferm.

[55] Chang B. L., Uman G. C., Linn L. S., JE Ware, Kane R. L., Dimond M. (1984). The effect of systematically varying components of nursing care on satisfaction in elderly ambulatory women. West J Nurs Res.

[56] Dufour E., Duhoux A., Contandriopoulos D. (2020). Measurement and Validation of Primary Care Nursing Indicators Based on a Wound Care Tracer Condition. J Nurs Care Qual.

[57] Blume K. S., Dietermann K., Kirchner-Heklau U., Winter V., Fleischer S., Kreidl L. M. (2021). Staffing levels and nursing-sensitive patient outcomes: Umbrella review and qualitative study. Health Serv Res.

[58] Galavote H. S., Zandonade E., Garcia A. C. P., Freitas P. S. S., Seidl H., Contarato P. C. (2016). The nurse’s work in primary health care. Esc Anna Nery.

[59] Sharma S. K., Rani R. (2020). Nurse-to-patient ratio and nurse staffing norms for hospitals in India: A critical analysis of national benchmarks. J Fam Med Prim Care.

[60] Pinto M. C., Silva L. S., Souza E. A. (2020). The Importance of Nursing Records Within the Audit Assessment Context. Arq Cien Saude UNIPAR.

[61] Baptista S. C. P. D., Juliani C. M. C. M., Silva e Lima S. G., Martin L. B., Silva K. A. B., Cirne M. R. (2021). Patient absenteeism in outpatient consultations: an integrative literature review. Rev Esc Enferm USP.

[62] Johns G., Taylor B., John A., Tan J. (2019). Current eating disorder healthcare services - the perspectives and experiences of individuals with eating disorders, their families and health professionals: systematic review and thematic synthesis. BJPsych Open.

[63] Senitan M., Alhaiti A. H., Lenon G. B. (2018). Factors contributing to effective referral systems for patients with non-communicable disease: evidence-based practice. Int J Diabetes Dev Ctries.

[64] Walker R. C., Tong A., Howard K., Palmer S. C. (2019). Patient expectations and experiences of remote monitoring for chronic diseases: Systematic review and thematic synthesis of qualitative studies. Int J Med Inform.

[65] Griffiths P., Richardson A., Blackwell R. (2012). Outcomes sensitive to nursing service quality in ambulatory cancer chemotherapy: Systematic scoping review. Eur J Oncol Nurs.

[66] Castro A. R., Abreu LDP, Lima LL, Araújo AF, Torres RAM, Silva MRF (2019). Nursing Consultation in the Outpatient Care of Youths. Rev Enferm UFPE On Line.

[67] Gilmore K. J., Corazza I., Coletta L., Allin S. (2023). The uses of Patient Reported Experience Measures in health systems: A systematic narrative review. Health Policy.

