# Supplementary material for: Quality of outpatient nursing care: a scoping review
Source: Rev Lat Am Enfermagem. 2025 Mar 31;33:e4524. doi: 10.1590/1518-8345.7028.4524 (PMC11960619; doi:10.1590/1518-8345.7028.4524)
Supplement: Translation

# Translation: Qualidade da assistência ambulatorial em enfermagem: uma revisão de escopo*

**DOI:** 10.1590/1518-8345.7028.4525

**Authors:** Bruna Noschang de Brum, Carlise Rigon Dalla Nora, Adriana Roese Ramos, Luciana Foppa, Deise Lisboa Riquinho

**Affiliations:** 1 Universidade Federal do Rio Grande do Sul, Escola de Enfermagem e de Saúde Coletiva, Porto Alegre, RS, Brasil.; 2 Bolsista da Coordenação de Aperfeiçoamento de Pessoal de Nível Superior (CAPES), Brasil.; 3 Hospital de Clínicas de Porto Alegre, Serviço de Enfermagem Ambulatorial, Porto Alegre, RS, Brasil.

**Keywords:** Assistência Ambulatorial, Atenção Secundária à Saúde, Enfermagem, Cuidados de Enfermagem, Indicadores de Qualidade em Assistência à Saúde, Qualidade da Assistência à Saúde

## Abstract

mapear as produções científicas nacionais e internacionais em saúde sobre a qualidade da assistência ambulatorial em enfermagem.

revisão de escopo orientada pela Colaboração *Joanna Briggs Institute*, realizada em 12 bases de dados e repositórios. Foram incluídos apenas artigos originais sobre a avaliação da qualidade da assistência ambulatorial em enfermagem. Não houve delimitação de tempo, metodologia ou idioma. Os dados foram mapeados e organizados por meio da descrição temática e estatística dos artigos. Esta revisão foi registrada na plataforma *Open Science Framework*.

foram encontrados 45 estudos publicados entre 1984 e 2021, originando 17 indicadores de qualidade da assistência ambulatorial de enfermagem, agrupados segundo a tríade de Donabedian: quatro de estrutura, sete de processo e seis de resultado. A área de cuidado predominante foi a oncologia. Dos estudos analisados, 55,3% tiveram foco nos pacientes. Os indicadores mais citados entre os estudos foram: educação permanente em saúde, organização do serviço, comunicação, coordenação do cuidado e processo de enfermagem.

o pequeno número de estudos nesse campo, em comparação com outras áreas da enfermagem, demonstra a pouca exploração do tema nas realidades nacional e internacional. Além disso, a diversidade de indicadores encontrada aponta para a falta de uniformização desses dados.

## Destaques

**(1)** Poucas pesquisas abordam a avaliação da qualidade ambulatorial em enfermagem. **(2)** Indicadores de qualidade mapeiam as dificuldades nos serviços de saúde. **(3)** Publicações sobre qualidade ambulatorial em enfermagem cresceram desde 2000. **(4)** A maioria das pesquisas avaliadas focou nos pacientes ambulatoriais. **(5)** O indicador de qualidade mais citado nos estudos foi Educação Permanente em Saúde.

## Introdução

A assistência ambulatorial refere-se aos cuidados de saúde realizados nesse nível de atenção, caracterizado especialmente pela não internação da clientela. Tais cenários de atendimento encontram-se em expansão devido às mudanças no padrão de atendimento de saúde, envelhecimento da população e maior prevalência de doenças crônicas^(1-3)^. Nesse sentido, busca-se aprimorar a qualidade do acesso aos cuidados de saúde, com a perspectiva de orientar a tomada de decisões^(4)^, visando adequá-las de modo a promover melhorias nos cuidados.

A avaliação da qualidade da assistência em saúde, amplamente utilizada como uma forma de categorizar os indicadores, busca contemplar diferentes perspectivas tanto de usuários e profissionais quanto de provedores de recursos de diferentes ordens^(5)^. Baseia-se na tríade de dimensões de Donabedian: estrutura, processos e resultados. A dimensão da estrutura engloba os recursos humanos, físicos, materiais e financeiros necessários para a prestação da assistência^(5)^. Já a dimensão do processo compreende as atividades desenvolvidas pelos profissionais de saúde e usuários, como diagnóstico, tratamento, relacionamento usuário-equipe^(5)^. A dimensão dos resultados, por sua vez, corresponde ao produto final da assistência, considerando a saúde, satisfação e expectativas dos usuários e profissionais^(5)^. Essa categorização permite inferências sobre os resultados dos cuidados, bem como sobre os processos que os antecedem ou o ambiente em que ocorrem^(5)^.

Destacam-se dois estudos disponíveis na literatura sobre indicadores de qualidade ambulatorial sensíveis à enfermagem^(6-7)^. A revisão da literatura evidenciou os seguintes indicadores, em um estudo*:* mudança na gravidade dos sintomas (*change in symptom severity*); força da aliança terapêutica (*strength of the therapeutic aliance*); utilização de serviços de saúde (*utilization of services*); satisfação do paciente (*client satisfaction*); redução do risco (*risk reduction*); aumento dos fatores de proteção (*increase in protective factors*) e estado funcional (*level of function/functional status*)^(6)^. E no outro, os indicadores citados foram: reconciliação de medicamentos (*medication reconciliation*); controle da hipertensão (*controlling high blood pressure*); avaliação da depressão (*depression assessment conducted*); avaliação da dor (*pain assessment and follow-up*) e readmissão do paciente (*hospital re-admissions*)^(7)^. Ambos enfatizam a necessidade de discussões mais aprofundadas sobre os indicadores de qualidade devido à complexidade das dimensões que os compõem, dificultando a determinação e aplicação de métricas de qualidade. Salienta-se que outras dimensões importantes relacionadas ao atendimento ambulatorial não foram contempladas pelos indicadores propostos. Entretanto, a utilização dos indicadores de qualidade na área da enfermagem se faz relevante, pois permite a visualização das contribuições que os cuidados de enfermagem trazem para os resultados dos pacientes.

Durante as buscas, encontrou-se apenas uma revisão de escopo referente ao tema da enfermagem ambulatorial^(8)^. Considerando os referidos aspectos, este estudo teve como foco indicadores de avaliação de desempenho sob o referencial de Dubois e não englobou pesquisas nos idiomas espanhol e português^(8)^, tornando a construção da presente revisão relevante para a produção de indicadores de qualidade que reflitam melhor a realidade brasileira e latino-americana.

Diante da relevância da enfermagem na assistência ambulatorial e por acreditar que faz parte das atribuições da enfermagem a preocupação com o desenvolvimento de práticas de cuidado de qualidade e em condições seguras, esta revisão tem por objetivo mapear as produções científicas nacionais e internacionais em saúde sobre a qualidade da assistência ambulatorial em enfermagem.

## Método

### Protocolo e registro

Trata-se de uma revisão de escopo, orientada pela Colaboração *Joanna Briggs Institute* (JBI)^(9)^. Para garantir a transparência e a qualidade, este estudo seguiu as diretrizes contidas no *Preferred Reporting Items for Systematic Reviews and Meta-Analyses extension for Scoping Reviews* (PRISMA-ScR)^(10)^. Esta revisão de escopo seguiu as seguintes etapas: definir e alinhar o objetivo e a pergunta; desenvolver e alinhar os critérios de inclusão com o objetivo e a pergunta; descrever a abordagem; planejar a busca de evidências; seleção, extração de dados e apresentação das evidências; procura pelas evidências; seleção das evidências; extração das evidências; análise das evidências; apresentação dos resultados; resumo das evidências em relação ao propósito da revisão, tirando conclusões e implicações das descobertas. Esta revisão teve o protocolo registrado na plataforma *Open Science Framework* (OSF)  sob o *Digital Object Identifier* (DOI) https://doi.org/10.17605/OSF.IO/6YP7N.

### Critérios de elegibilidade

Os critérios de inclusão foram artigos originais cujos objetos abordassem a avaliação da qualidade da assistência ambulatorial em enfermagem, motivada pela maior rigidez e robustez metodológica associada a esse tipo de estudo. Assim, foram incluídos estudos com delineamento quantitativo, qualitativo ou mistos. Elencaram-se estudos que tinham como participantes enfermeiros envolvidos na assistência ambulatorial em serviços de saúde públicos ou privados, pacientes atendidos por enfermeiros em ambiente ambulatorial especializado ou estudos que descreviam atividades assistenciais de enfermagem ambulatorial.

Os critérios de exclusão foram: estudos teóricos, de revisão, metodológicos, de caso, editoriais, relatos de experiência, dissertações e teses. Não houve limite temporal ou de idioma, visando a inclusão do maior número possível de estudos.

### Fontes de informação

Os estudos foram selecionados em diferentes repositórios e bases de dados: Literatura Latino-Americana e do Caribe em Ciências da Saúde (LILACS), Índice Bibliográfico Espanhol de Ciências da Saúde (IBECS), *Medical Literature Analysis and Retrieval System Online* (Medline) e Base de Dados de Enfermagem (BDENF) via Biblioteca Virtual em Saúde (BVS); *Scientific Electronic Library Online* (SciELO); PubMed; *Web of Science Core Collection* via *Clarivate;* Embase e Scopus via Elsevier; *Cumulative Index to Nursing and Allied Health Literature* (CINAHL) via Ebsco. A busca na literatura cinzenta foi conduzida por meio da ferramenta *Google* Acadêmico.

### Busca na literatura

A busca pelos estudos foi realizada em 22 de outubro de 2021 e atualizada em 30 de abril de 2022.

Tanto a questão de investigação quanto a estratégia de busca implementada neste estudo foram elaboradas a partir do mnemônico PCC, sendo: P: População – isto é, publicações científicas nacionais e internacionais sobre qualidade da assistência em enfermagem; C: Conceito – qualidade da assistência em enfermagem; e C: Contexto – serviços ambulatoriais. Assim, a questão norteadora elaborada foi: “o que as publicações científicas nacionais e internacionais dizem sobre a qualidade da assistência em enfermagem em serviços ambulatoriais?”.

A estratégia de busca foi definida considerando os Descritores em Ciências da Saúde (DeCS) e *Medical Subject Headings* (MeSH) somente em inglês. Os descritores selecionados foram: “assistência ambulatorial”; “cuidados de enfermagem”; ” atenção secundária à saúde”; “qualidade da assistência à saúde”, mantendo o operador booleano *AND*, respeitando as peculiaridades e características de cada base de dados conforme Figura 1.

**Figura 1 -:** Estratégias de busca referentes às bases de dados pesquisadas. Rio Grande do Sul, Brasil, 2023

**Bases de dados**	**Estratégia de busca**
LILACS, IBECS, Medline e BDENF (via Biblioteca Virtual em Saúde)	*(“Nursing Care”) AND (“Secondary Care”) AND (“Quality of Health Care”)* *(“Nursing Care”) AND (“Ambulatory Care”) AND (“Quality of Health Care”)*
CINAHL (via Ebsco)	*(“Nursing Care”) AND (“Secondary Care”) AND (“Quality of Health Care”)* *(“Nursing Care”) AND (“Ambulatory Care”) AND (“Quality of Health Care”)*
SciELO	*(Nursing Care) AND (Secondary Care) AND (Quality of Health Care)* *(Nursing Care) AND (Ambulatory Care) AND (Quality of Health Care)*
PubMed	*(Nursing Care) AND (Ambulatory Care) AND (Quality of Health Care)* *(“Nursing Care”) AND (“Ambulatory Care”)*
Embase (via Elsevier)	*nursing AND care AND ‘secondary health care’ AND ‘health care quality’* *nursing care’ AND ‘ambulatory care’ AND ‘health care quality’*
*Web of Science Core Collection* (via *Clarivate* )	*(Nursing Care) AND (Ambulatory Care) AND (Quality of Health Care)* *(Nursing Care) AND (Secondary Care) AND (Quality of Health Care)* *(Nursing Care) AND (Ambulatory Care)* *(“Nursing Care”) AND (“Secondary Care”)* *(“Ambulatory Care”) OR (“Secondary Care”) AND (“Nursing Care”)*
Scopus (via *Elsevier* )	*(“Nursing Care”) AND (“Ambulatory Care”) AND (“quality off health care”)* *(“Nursing Care”) AND (“Secondary Care”) AND (“quality off health care”)*
*Google* Acadêmico	*“Nursing Care” AND “Ambulatory Care” AND “Quality of Health Care”*

### Seleção dos estudos

Para a organização dos artigos, eles foram armazenados no gerenciador de referências *Zotero* ^©^. Quanto à seleção dos estudos, os resultados das buscas foram analisados por duas pesquisadoras, de forma independente, utilizando-se de formulários do *Google Forms* ^©^ e das Planilhas *Google* ^©^. As discordâncias foram resolvidas por consenso ou com a presença de um terceiro pesquisador para avaliação. Em outras palavras, os pesquisadores confrontaram os resultados das buscas realizadas, verificando as diferenças dos achados, sempre visando incluir o maior número de estudos possível.

### Extração e análise de dados

Para a etapa da extração dos dados, utilizou-se um formulário estruturado na plataforma *Google Forms* ^©^ para identificar e descrever os seguintes itens: autor; ano de publicação; país; periódico; participantes; abordagem realizada e principais resultados que estivessem relacionados à qualidade da assistência ambulatorial em enfermagem, separando-os segundo as dimensões estrutura, processos e resultados.

## Resumo dos resultados

Os dados foram coletados a partir dos resultados dos 45 estudos selecionados, destacando a relação de cada estudo com a qualidade da assistência. A extração desses dados permitiu mapeá-los, interpretá-los e realizar análise numérica básica da extensão, natureza e distribuição dos estudos incorporados na revisão. Em seguida, realizou-se o agrupamento por temática e a descrição estatística dos resultados em Planilhas *Google* ^©^, com o intuito de apresentar uma visão geral de todo o material. Após a organização através da descrição temática dos resultados, eles foram agrupados em categorias segundo a tríade estrutura, processos e resultados proposta por Donabedian^(5)^, utilizando quadros de acordo com a sua relevância.

O processo de coleta de dados e de seleção dos estudos será apresentado nos resultados deste artigo por meio de um fluxograma que segue as diretrizes PRISMA-ScR^(10)^.

## Resultados

Identificaram-se 1.530 estudos provenientes de bases de dados e repositórios. Após a identificação e exclusão de estudos duplicados, restaram 794 estudos. Estes foram avaliados por meio da leitura dos títulos e resumos, excluindo-se aqueles que não estavam de acordo com a temática, resultando em 225 estudos para avaliação integral do texto. Após a leitura completa, 180 estudos foram excluídos: 119 não se adequavam ao tema da revisão, 48 não atendiam aos critérios de inclusão e 13 não puderam ser recuperados.

Os estudos foram pré-selecionados a partir da leitura dos títulos e resumos, e a amostra final foi alcançada com base na leitura dos artigos na íntegra, conforme fluxograma apresentado na Figura 2.

**Figura 2 -:** Fluxograma da seleção dos estudos que compõem a pesquisa adaptado do PRISMA ScR^(10)^. Rio Grande do Sul, Brasil, 2023

A partir da seleção dos artigos resultantes das buscas nas bases de dados e repositórios a amostra desta revisão foi composta por 45 artigos publicados entre 1984 e 2021. A ampliação do conhecimento nessa área de atuação pode ser identificada pelo aumento no número de publicações a partir do ano 2000, mais especificamente nos anos de 2012 e 2018, pois apresentam maior número de estudos em comparação com os demais anos.

A fim de facilitar a apresentação, optou-se por organizar os dados mais relevantes de cada artigo em um quadro, conforme apresentado na Figura 3.

**Figura 3 -:** Caracterização dos artigos segundo autor, ano de publicação, país de realização do estudo, idioma, abordagem, amostra e área de cuidado. Rio Grande do Sul, Brasil, 2023

**Citação**	**Ano/País/Idioma**	**Abordagem**	**Amostra**	Área de Cuidado
Cunha, et al., 2021 ^(11)^	2021 Brasil Português	Quantitativo (longitudinal)	Pacientes	Cardiologia
Seibert, et al., 2020 ^(12)^	2020 Alemanha Alemão	Quantitativo (transversal)	Indicadores de qualidade	Geriatria
Ameel, et al., 2020 ^(13)^	2020 Finlândia Inglês	Qualitativo (descritivo)	Registros de enfermagem*	Psiquiatria
Zendrato; Hariyati; Afifah, 2019 ^(14)^	2019 Indonésia Inglês	Quantitativo (descritivo)	Pacientes	Policlínica
Errico, et al., 2018 ^(15)^	2018 Brasil Inglês	Quantitativo (transversal)	Registros de enfermagem*	Obstetrícia
Heale, et al., 2018 ^(16)^	2018 Canadá Inglês	Quantitativo (transversal)	Registros de enfermagem*	Endocrinologia
Connor, et al., 2018 ^(17)^	2018 EUA Inglês	Quantitativo (descritivo)	Enfermeiros ^†^	Cardiologia pediátrica
Seabra; Amendoeira; Sá, 2018 ^(18)^	2018 Portugal Inglês	Quantitativo (transversal)	Pacientes	Psiquiatria
Anderson, et al., 2018 ^(19)^	2018 EUA Inglês	Qualitativo (descritivo)	Profissionais da saúde Familiares de pacientes	Cardiologia pediátrica
Silva, et al., 2018 ^(20)^	2018 Brasil Inglês	Qualitativo (descritivo)	Pacientes	Estomaterapia
Calvo; Sepulveda-Carrillo, 2017 ^(21)^	2017 Colômbia Inglês	Quantitativo (transversal)	Pacientes	Oncologia
Ye, et al., 2016 ^(22)^	2016 EUA Inglês	Quantitativo (longitudinal)	Pacientes	Pediatria
Selvin, et al., 2016 ^(23)^	2016 Suíça Inglês	Qualitativo (descritivo)	Pacientes	Psiquiatria
Macedo, et al., 2016 ^(24)^	2016 Brasil Inglês	Qualitativo (descritivo)	Enfermeiros ^†^	SAE HIV/Aids
Vessey, et al., 2015 ^(25)^	2015 EUA Inglês	Qualitativo (descritivo)	Pacientes	Pediatria
Vanderboom; Thackeray; Rhudy, 2015 ^(26)^	2015 EUA Inglês	Qualitativo (descritivo)	Enfermeiros ^†^	Policlínica
Tuna, et al., 2015 ^(27)^	2015 Turquia Inglês	Quantitativo (descritivo)	Enfermeiros ^†^	Oncologia
Komatsu; Yagasaki, 2014 ^(28)^	2014 Japão Inglês	Qualitativo (transversal)	Enfermeiros ^†^	Oncologia
Hammelef, et al., 2014 ^(29)^	2014 EUA Inglês	Quantitativo (transversal)	Pacientes	Oncologia
Font Difour, et al., 2014 ^(30)^	2014 Cuba Espanhol	Quantitativo (descritivo)	Pacientes e	Oncologia
Armes, et al., 2014 ^(31)^	2014 Inglaterra Inglês	Quantitativo (transversal)	Pacientes	Oncologia
Van Den Bussche, et al., 2013 ^(32)^	2013 Alemanha Alemão	Quantitativo (descritivo)	Profissionais de saúde	Psiquiatria
Palese, et al., 2013 ^(33)^	2013 Itália Inglês	Quantitativo (transversal)	Registros de enfermagem*	Oncologia
Callen, et al., 2013 ^(34)^	2013 Austrália Inglês	Misto	Enfermeiros ^†^	Reumatologia
Williams, et al., 2012 ^(35)^	2012 Reino Unido Inglês	Quantitativo (descritivo)	Pacientes	Cardiologia
Pinto, et al., 2012 ^(36)^	2012 Brasil Inglês	Qualitativo (descritivo)	Enfermeiros ^†^	Sem descrição
Pfeiffer, et al., 2012 ^(37)^	2012 EUA Inglês	Misto	Enfermeiros ^†^	Sem descrição
Larsson, et al., 2012 ^(38)^	2012 Suécia Inglês	Qualitativo (descritivo)	Pacientes	Reumatologia
Kamimura, et al., 2012 ^(39)^	2012 EUA Inglês	Qualitativo (descritivo)	Enfermeiros ^†^	Oncologia
Friese; Manojlovich, 2012 ^(40)^	2012 EUA Inglês	Misto	Enfermeiros ^†^	Oncologia
Hjoerleifsdottir; Hallberg; Gunnarsdottir, 2010 ^(41)^	2010 Islândia Inglês	Quantitativo (transversal)	Pacientes	Oncologia
Skrutkowski, et al., 2008 ^(42)^	2008 Canadá Inglês	Quantitativo (intervencional)	Pacientes	Oncologia
Rootmensen, et al., 2008 ^(43)^	2008 Holanda Inglês	Quantitativo (intervencional)	Pacientes	Pneumologia
Sisk, et al., 2006 ^(44)^	2006 EUA Inglês	Quantitativo (intervencional)	Pacientes	Cardiologia
Fonseca; Gutiérrez; Adami, 2006 ^(45)^	2006 Brasil Português	Quantitativo (descritivo)	Pacientes	Oncologia
Mohrmann, et al., 2005 ^(46)^	2005 Alemanha Alemão	Quantitativo (descritivo)	Pacientes	Sem descrição
Gesell; Gregory, 2004 ^(47)^	2004 EUA Inglês	Quantitativo (descritivo)	Pacientes	Oncologia
Cusack; Jones-Wells; Chisholm, 2004 ^(48)^	2004 EUA Inglês	Quantitativo (descritivo)	Pacientes	Oncologia
Arthur; Clifford, 2004 ^(49)^	2004 Reino Unido Inglês	Quantitativo (transversal)	Pacientes	Reumatologia
Zink, et al., 2000 ^(50)^	2000 Alemanha Alemão	Quantitativo (descritivo)	Registros de enfermagem*	Sem descrição
Oermann; Templin, 2000 ^(51)^	2000 EUA Inglês	Qu antitativo (transversal)	Pacientes	Sem descrição
Oermann; Dillon; Templin, 2000 ^(52)^	2000 EUA Inglês	Quantitativo (descritivo)	Pacientes	Sem descrição
Sanna, 1993 ^(53)^	1993 Brasil Português	Qualitativo (descritivo)	Familiares de pacientes	Pediatria
Silva, 1985 ^(54)^	1985 Brasil Português	Quantitativo (intervencional)	Pacientes	Endocrinologia
Chang, et al., 1984 ^(55)^	1984 EUA Inglês	Quantitativo (intervencional)	Pacientes	Geriatria

*Registros de enfermagem = Engloba todos os tipos de documentações preenchidas pela equipe de enfermagem; ^†^Enfermeiros = Pela diversidade de locais de estudo e diferentes organizações da categoria de enfermagem, optou-se por padronizar a população como enfermeiros, entretanto engloba diversos profissionais desta categoria

Com a análise da Figura 3, conclui-se que 93,3% dos artigos foram publicados a partir do ano 2000, 80% dos artigos encontram-se em língua inglesa^(13-29,31,33-44,47-49,51-52,55)^ e 66,7% apresentam abordagem metodológica quantitativa^(11-12,14-18,21-22,27,29-35,37,40-55)^. Das 19 localidades identificadas, Estados Unidos^(17,19,22,25-26,29,37,39-40,44,47-48,51-52,55)^ foi o país com mais artigos publicados 33,3%, seguidos por 12,8% no Brasil^(11,15,20,24,36,45,53-54)^ e 8,9% na Alemanha^(12,32,46,50)^.

Em relação à amostra dos estudos, 55,3% tiveram foco nos pacientes^(11,14,18,20-23,25,29,31,35,38,41-49,51-52,54-55)^, seguido por 23,4% cujo enfoque foi em enfermeiros^(17,24,26-28,30,34,36-37,39-40)^, 10,6% em registros de enfermagem^(13,15-16,33,50)^, 4,3% em familiares de pacientes^(19,53)^, 4,3% em profissionais de Saúde^(19,32)^ e 2,1% em indicadores de qualidade^(12)^. A área de cuidado predominante entre os artigos foi a de oncologia^(21,27-31,33,39-42,45,47-48)^, com 31,1% das publicações.

A partir do agrupamento por temática dos artigos desta revisão, considerando a questão de pesquisa e objetivo proposto, emergiram 17 indicadores que interferem na qualidade da assistência de enfermagem, apresentados na Figura 4. A distribuição desses indicadores ocorreu da seguinte forma: 43,2% na dimensão processo^(12-23,25-28,30-35,37-39,41-54)^, 30,3% na dimensão resultado^(11,18,20-24,28-29,31-32,35-47,49,51-55)^ e 26,5% na dimensão estrutura^(13-14,16-22,24,27,34-37,39-41,43-49,51-52,55)^.

**Figura 4 -:** Indicadores de qualidade do atendimento ambulatorial em enfermagem segundo a tríade estrutura, processos e resultados de Donabedian^(5)^. Rio Grande do Sul, Brasil, 2023

**Estrutura**	**Processo**	**Resultados**
Dimensionamento de pessoal ^(27 , 36 , 39 - 40 , 45)^	Competências de enfermagem ^(30 - 31 , 34 - 35 , 37 , 40 , 46)^	Autocuidado ^(11 , 20 - 21 , 23 , 31 , 42 - 44 , 52 , 54)^
Educação permanente em saúde ^(14 , 16 , 19 , 24 , 36 - 37 , 43 , 45 - 46 , 48 - 49 , 51 - 52 , 55)^	Comunicação ^(14 , 16 , 22 - 23 , 32 , 37 - 41 , 51 - 52)^	Relacionamento enfermeiro-equipe ^(24 , 32 , 37 , 39 - 40)^
Infraestrutura ^(14 , 16 , 22 , 36 , 39 - 41 , 45 , 47)^	Coordenação do cuidado ^(15 - 17 , 25 - 26 , 28 , 32 , 40 , 42 , 44 , 47 , 49)^	Relacionamento enfermeiro-paciente ^(22 - 23 , 28 , 38 , 45 , 47 , 49 , 51 - 53 , 55)^
Organização do serviço ^(13 , 16 , 20 , 22 , 34 - 36 , 39 , 41 , 44 , 46 - 47 , 52)^	Cuidados de enfermagem ^(13 , 16 , 18 , 20 , 27 , 41 , 43 , 46 , 48 , 53)^	Satisfação do paciente ^(18 , 35 , 38 , 41 , 45 - 46 , 49 , 51 - 53 , 55)^
	Diagnóstico ^(12 , 15 , 18)^	Satisfação do profissional ^(36 - 37 , 39)^
	Educação em saúde ^(17 , 21 , 41 - 45 , 47 , 51 - 52 , 54)^	Saúde mental ^(18 , 21 , 28 - 29 , 35 , 42 , 47)^
	Processo de enfermagem ^(13 - 16 , 19 , 26 , 30 , 31 , 46 - 48 , 50)^	

### Indicadores de qualidade da dimensão estrutura

Os estudos incluídos nesta categoria foram quatro indicadores: dimensionamento de pessoal, educação permanente em saúde, infraestrutura e organização do serviço.

O dimensionamento de pessoal^(27,36,39-40,45)^ envolve recursos humanos, carga de trabalho, dimensionamento das equipes de saúde, número de pacientes por profissional de acordo com as necessidades de cuidado de cada paciente e o nível de expertise exigido do profissional, além da disponibilidade e acessibilidade dos profissionais de saúde.

A educação permanente em saúde^(14,16,19,24,36-37,43,45-46,48-49,51-52,55)^ refere-se às atividades educativas direcionadas aos profissionais de saúde, incluindo capacidade técnica, conhecimento teórico e técnico, experiência profissional, formação continuada, *feedback* e práticas baseadas em evidências.

A infraestrutura^(14,16,22,36,39-41,45,47)^ está relacionada às instalações físicas dos serviços de saúde, à disponibilidade de recursos materiais e da comunidade, ao tempo de espera para atendimento e à acessibilidade dos serviços de saúde.

A organização do serviço^(13,16,20,22,34-36,39,41,44,46-47,52)^ compreende aspectos organizacionais das entidades de saúde, como acesso, equipe, modelo assistencial vigente, atividades gerenciais, processos de auditoria e tecnologias da informação em saúde.

### Indicadores de qualidade da dimensão processo

Os estudos incluídos nesta categoria apresentaram sete indicadores: competências de enfermagem, comunicação, coordenação do cuidado, cuidados de enfermagem, diagnóstico, educação em saúde e processo de enfermagem.

As competências de enfermagem^(30-31,34-35,37,40,46)^ englobam atividades relacionadas às competências necessárias aos enfermeiros como: liderança, gestão da equipe e dos recursos materiais, trabalho em equipe, gerenciamento de conflitos, atividades administrativas, segurança do paciente, supervisão e gestão das atividades de cuidado.

A comunicação^(14,16,22-23,32,37-41,51-52)^ envolve a capacidade do enfermeiro de transmitir conhecimentos aos pacientes, familiares e comunidade, garantindo que as orientações sejam compreendidas pelos pacientes e que os profissionais entendam as suas necessidades em uma troca de informações. Para isso, é necessário atenção, empatia, sensibilidade, assertividade e respeito, facilitando, assim, a comunicação eficaz.

A coordenação do cuidado^(15-17,25-26,28,32,40,42,44,47,49)^ refere-se às atividades desenvolvidas em colaboração com outros profissionais de saúde, como encaminhamento, continuidade do cuidado de acordo com as necessidades do paciente, agendamento de consultas e exames, renovação de prescrições, acompanhamento do paciente e da família.

Os cuidados de enfermagem^(13,16,18,20,27,41,43,46,48,53)^ envolvem as atividades de cuidado direto ao paciente, como intervenções de enfermagem, avaliação da intensidade do cuidado, características da equipe e dos pacientes, triagem e cuidado integral. O diagnóstico^(12,15,18)^ refere-se às patologias apresentadas pelos pacientes conforme o Código Internacional de Doenças (CID).

A educação em saúde^(17,21,41-45,47,51-52,54)^ abrange atividades educativas destinadas à orientação de pacientes e da comunidade sobre agravos de saúde e informações para a promoção, prevenção e recuperação da saúde. Os artigos mencionam o desenvolvimento de atividades de aconselhamento sobre hábitos saudáveis, informações sobre doenças e procedimentos, prevenção de eventos adversos e a redução no desperdício de recursos materiais.

O processo de enfermagem^(13-16,19,26,30,31,46-48,50)^ refere-se a todas as atividades que envolvem as cinco etapas do processo de enfermagem: coleta de dados, diagnóstico, planejamento, implementação e avaliação, incluindo o registro de enfermagem.

### Indicadores de qualidade da dimensão resultado

Os estudos incluídos nesta categoria apresentaram seis indicadores: autocuidado, relacionamento enfermeiro-equipe, relacionamento enfermeiro-paciente, satisfação do paciente, satisfação do profissional e saúde mental.

O autocuidado^(11,20-21,23,31,42-44,52,54)^ está relacionado à participação do paciente no processo do cuidado e no manejo dos sinais e sintomas. A iniciativa do paciente e dos familiares para a promoção e manutenção da saúde, adesão ao tratamento e o desenvolvimento de atividades que incentivem o paciente a tornar-se agente ativo e corresponsável pelo próprio cuidado fazem parte das estratégias citadas nos estudos.

O relacionamento enfermeiro-equipe^(24,32,37,39-40)^ refere-se à relação do enfermeiro com seus colegas de trabalho, seja profissionais de enfermagem ou de outras categorias. Envolve respeito, compreensão, confiança, comunicação, disponibilidade, comportamento, autonomia e manejo de situações de conflitos.

O relacionamento enfermeiro-paciente^(22-23,28,38,45,47,49,51-53,55)^ relaciona-se com a dinâmica de comportamento entre enfermeiros e pacientes. O estímulo à autonomia, o acompanhamento do paciente, encontros baseados na cordialidade, empatia, escuta ativa, sentimentos de compreensão, aceitação e sensibilidade, resultam em sensação de confiança e segurança.

A satisfação do paciente^(18,35,38,41,45-46,49,51-53,55)^ está relacionada à percepção do paciente sobre o resultado final do cuidado. O nível de satisfação com o cuidado prestado, os valores, a participação, as condutas, as informações relacionadas à saúde, o vínculo com o profissional e o ambiente influenciam a visão do paciente em relação à experiência do cuidado.

A satisfação do profissional^(36-37,39)^ refere-se à percepção dos profissionais sobre as suas atividades de trabalho. O nível de satisfação ou insatisfação profissional é influenciado pelas atividades exercidas, pela dinâmica da equipe de trabalho e pelas instalações de saúde.

A saúde mental^(18,21,28-29,35,42,47)^ engloba as necessidades emocionais dos pacientes e dos profissionais. O grau de sofrimento psicológico pode ser influenciado por conflitos e pelo nível de confiança entre profissionais de saúde e pacientes ou entre membros da equipe de saúde. O desenvolvimento de atividades educativas relacionadas ao bem-estar mental e o encaminhamento para suporte emocional, de acordo com as necessidades dos indivíduos, podem amenizar esses problemas.

## Discussão

Os resultados apresentados nesta revisão auxiliam no mapeamento da produção do conhecimento na área de enfermagem ambulatorial especializada. Grande parte dos estudos desta revisão corresponde à realidade internacional de atuação da enfermagem, o que pode dificultar a aplicação na realidade brasileira. A maioria dos artigos teve como população os pacientes atendidos pelas equipes de enfermagem, evidenciando a importância do paciente no desenvolvimento dos níveis de qualidade dos serviços^(11,14,18,20-23,25,29,31,35,38,41-49,51-52,54-55)^. Ainda que determinadas especialidades de enfermagem que atuam no atendimento ambulatorial tenham uma produção maior de estudos, como, por exemplo, a oncologia^(21,27-31,33,39-42,45,47-48)^, tiveram os estudos desenvolvidos em conjunto com o ambiente hospitalar^(56)^, o que dificulta determinar se os resultados traduzem bem a realidade ambulatorial.

A enfermagem tem papel importante na assistência ambulatorial, implementando cuidados centrados no paciente e na família. Assim, os indicadores de qualidade devem refletir a natureza das intervenções e suas preocupações^(8)^. Em nossas análises, encontramos quatro indicadores de saúde relacionados à estrutura – dimensionamento de pessoal, educação permanente em saúde, infraestrutura, organização do serviço; sete indicadores de processo – competências de enfermagem, comunicação, coordenação do cuidado, cuidados de enfermagem, diagnóstico, educação em saúde, processo de enfermagem; e seis de resultados – autocuidado, relacionamento enfermeiro-equipe, relacionamento enfermeiro-paciente, satisfação do paciente, satisfação do profissional, saúde mental. A literatura tem indicado maior foco na utilização de indicadores de processo para melhor a qualidade dos serviços, pois estes apresentam forte associação com os resultados^(56)^. Observa-se que a melhoria de indicadores nessas duas dimensões pode gerar impactos significativos nos serviços de saúde^(56)^.

A conexão entre os cuidados de enfermagem e o bem-estar dos pacientes é complexa. Não cuidar adequadamente da força de trabalho de enfermagem pode impactar profundamente na qualidade dos cuidados, repercutindo negativamente na saúde dos pacientes^(8)^. Cargas de trabalho excessivas, falta de estrutura organizacional nos serviços de saúde e a rotatividade dos profissionais compromete o vínculo com a comunidade e influenciam na qualidade da assistência^(56-57)^. Além do mais, mudanças constantes de trabalhadores ocasionam sobrecarga nas equipes, pois exigem o constante treinamento de novos membros, aumentando os custos e fragilizando os processos de trabalho^(57)^. Um baixo número de pacientes por enfermeiro está sendo associado a melhores resultados^(8)^ de saúde e locais onde a proporção de pacientes para enfermeiros é baseada nos cuidados de enfermagem que fornecem práticas assistenciais de melhor qualidade e baseadas em evidências^(8)^. Na Califórnia, o *National Nurses United* utiliza um modelo de proporções fixas de enfermeiros para pacientes, estabelecendo níveis obrigatórios de profissionais dependentes do ambiente e das condições de saúde dos pacientes^(58)^.

As funções de gestão do enfermeiro por vezes é reduzida a atividades de natureza burocrática e organizacional, levando à sobrecarga do profissional que deve gerenciar a produção do cuidado assistencial e o trabalho administrativo burocrático de gestão de pessoal e insumos^(57)^. Apesar da importância da organização do serviço de saúde para evitar prejuízos na continuação do cuidado do paciente^(59-60)^, a burocratização e mecanização do trabalho do enfermeiro põem em risco a saúde dos pacientes^(57)^, podendo levar ao abandono do cuidado direto que, por vezes, é delegado aos técnicos e auxiliares de enfermagem^(57)^.

Alguns problemas de saúde podem exigir tratamento especializado e necessitar de articulação entre os níveis de atenção primários e secundários. A coordenação entre os diferentes níveis de atendimento pode ser difícil; a falta de confiança e de conhecimento dos profissionais podem culminar no encaminhamento desnecessário de pacientes para os serviços especializados. No entanto, esses serviços frequentemente encontram-se com poucos recursos disponíveis e sobrecarregados^(61)^. Os encaminhamentos são parte importante do manejo dos pacientes, pois garantem a continuidade dos cuidados^(62)^. Um bom matriciamento realizado pelos serviços especializados aumentaria a capacidade técnica e a confiança dos profissionais para lidar com esses casos, melhorando a resolutividade da atenção primária, evitando encaminhamentos desnecessários^(61)^.

Alguns estudos relatam que as dificuldades de acesso aos serviços de saúde são um dos principais motivos para o absenteísmo nas consultas, o que prejudica o acompanhamento e a continuidade do cuidado^(60)^. Sugere-se que, à medida que o percentual de continuidade aumenta, os resultados de saúde melhoram^(56)^, pois a continuidade promove a construção de uma relação de confiança entre o profissional e o paciente, o que favorece a comunicação eficaz e melhora a adesão ao tratamento^(56)^.

Estudos que abordam a comunicação representaram uma parte da nossa amostra. Os pacientes identificam a comunicação como um indicador de alta qualidade dos cuidados de saúde; uma boa comunicação entre profissionais e pacientes se traduz em melhores resultados^(63)^. Os atributos da comunicação com o paciente devem incluir a capacidade de ouvir, o respeito, a cortesia, a explicação clara e a linguagem adequada^(63)^. Além disso, uma comunicação adequada também deve ocorrer entre os profissionais, bem como entres as instituições de saúde^(63)^.

Alguns estudos discorrem sobre os registros de enfermagem, que compreendem toda a documentação preenchida pelos enfermeiros, sendo essenciais para garantir a qualidade da assistência e a segurança do paciente^(59)^. A documentação das etapas do processo de enfermagem – anamnese e exame físico, diagnóstico de enfermagem, prescrição de enfermagem e avaliação^(64)^ – é um instrumento importante para a continuidade do cuidado, servindo como registo das atividades exercidas pela equipe, assegurando o fornecimento de informações importantes sobre a assistência e mantendo arquivados dados relevantes no processo de auditoria^(59)^. Entretanto, os registros de enfermagem foram apontados como um dos campos com maior déficit de qualidade do processo de enfermagem no Brasil. Ainda que a sua importância seja reconhecida, a produção de registros adequados encontra diversos limitadores na prática assistencial, como a carência de profissionais, excesso de trabalho e falta de conhecimento teórico^(59)^.

Entre as outras competências necessárias aos enfermeiros, destaca-se a importância do conhecimento técnico-científico, das habilidades de relacionamento e da capacidade de gestão administrativa, assistencial e de pessoal. Manter um relacionamento adequado com os pacientes, familiares e membros da equipe de saúde envolve demonstrar comprometimento, envolvimento e postura ética^(57)^. Já a liderança de enfermagem é indispensável para a criação de bons ambientes de trabalho e tem forte ligação com a satisfação profissional e com a melhora da saúde mental. Entretanto, sua utilização não se detém apenas à gestão da equipe de saúde, sendo também de grande relevância para a promoção de cuidados preventivos.

Reconhecer que os próprios pacientes são os melhores e mais qualificados para fornecer informações sobre o que é importante no cuidado e nas interações com os profissionais de saúde^(65)^ tem grande impacto na qualidade e na satisfação dos pacientes, já que as evidências apontam para a associação entre experiência positiva, melhores desfechos e maior adesão ao tratamento^(65)^. Os dados de experiência do paciente, que normalmente são obtidos por meio de pesquisas de satisfação, podem ser utilizados como informações de desempenho em todos os níveis do sistema de saúde, pois fornecem dados robustos sobre a assistência^(65-66)^.

Já o indicador educação em saúde influencia nos resultados do paciente e é um componente-chave da prática da enfermagem^(56)^, pois aumenta a capacidade de autogestão do paciente, levando a menores riscos de agudização das condições de saúde a curto e a longo prazo^(56)^. Embora a importância da educação em saúde para a qualidade do cuidado esteja relatada na literatura, poucos artigos detalham as atividades educativas implementadas ou avaliam o impacto dessas intervenções nos pacientes.

A educação também é um fator importante para a capacitação dos profissionais de saúde. A Organização Pan-Americana da Saúde (OPAS) já alertou sobre a formação de profissionais de saúde sob um currículo universitário pautado em paradigmas curativos, hospitalocêntrico e de conhecimentos de saúde fragmentado, valorizando as especialidades e deixando de lado a compreensão global do ser humano e dos processos de saúde e doença^(57)^. Por isso, a educação permanente é uma ferramenta importante para a qualificação do trabalho do enfermeiro e deve considerar cada contexto e exigências de trabalho^(57)^.

A prática baseada em evidências esteve presente nos estudos analisados. Esse método é definido como uma abordagem para resolver problemas de saúde e melhorar a tomada de decisão, conduzida por uma busca das melhores e mais recentes evidências, que incluem a experiência clínica, a avaliação e as preferências do paciente dentro de um contexto de saúde. Os estudos indicaram que a prática baseada em evidências aumenta a qualidade dos sistemas de saúde, melhora os resultados dos pacientes, diminui os custos e promove maior satisfação^(62)^. Já o indicador autocuidado está fortemente inserido no escopo da prática da enfermagem, pois os enfermeiros oferecem rotineiramente esse serviço através de práticas de educação em saúde^(67)^.

Os resultados provenientes desta revisão contribuem para o avanço do conhecimento científico na área da enfermagem em saúde coletiva, especialmente para a qualificação da assistência ambulatorial. As evidências coletadas auxiliam no preenchimento da lacuna de conhecimento sobre os indicadores de qualidade ambulatorial em enfermagem, visto que os enfermeiros têm papel importante na gestão do cuidado. Entretanto, parece haver barreiras para a tradução dos resultados das pesquisas para a prática assistencial. Caminhos futuros de pesquisa nesta área podem investigar quais são essas barreiras, visto que estes já são amplamente utilizados na assistência hospitalar há muito tempo.

Este estudo teve como limitação a dificuldade em obter pesquisas específicas na área de enfermagem ambulatorial especializada. Apesar do tema da qualidade ambulatorial em enfermagem não ser recente na literatura, a produção de pesquisas nesse nível de atenção ainda é pequena comparada com o grande número de estudos produzido na área hospitalar e da atenção primária. Além disso, a dificuldade em obter artigos primários focados nos cuidados ambulatoriais especializados limitou a possibilidade de realizar inferências. Seria necessário um conjunto maior de dados primários para garantir uma análise mais aprofundada. Como resultado, as relações discutidas neste artigo foram obtidas, em sua maioria, de estudos que tinham como foco principal outras configurações ambulatoriais.

A utilização de diferentes descritores ou bases de indexação não incluídas nesta revisão poderiam resultar na obtenção de outros estudos. Além disso, a inclusão de apenas artigos originais, a não utilização dos descritores em outros idiomas – como português e espanhol – ou de sinônimos pertinentes, bem como a omissão de outros descritores – como o *Thesaurus* do Cinahl e o *Emtree* da Embase – podem ter gerado um viés na identificação dos estudos. Logo, os autores reconhecem que importantes pesquisas publicadas podem ter sido omitidas usando a nossa estratégia de busca.

Os estudos mapeados demonstram desigualdade em publicações nacionais e internacionais sobre o tema, sendo este pouco explorado na realidade brasileira, além da existência de uma diversidade de indicadores que interferem na qualidade do cuidado de enfermagem. Portanto, parece haver uma lacuna no conhecimento científico no que tange à uniformização dos indicadores de qualidade da assistência ambulatorial de enfermagem.

## Conclusão

O mapeamento das produções científicas nacionais e internacionais em saúde sobre a qualidade do atendimento ambulatorial em enfermagem mostrou pouca exploração do tema, sobretudo se comparada com outras áreas de conhecimento da enfermagem ambulatorial, tanto em âmbito nacional quanto internacionalmente. A partir do agrupamento por temática dos dados resultantes da análise, emergiram 17 indicadores que interferem na qualidade da assistência de enfermagem, estando em sua maioria na dimensão processo. Os indicadores mais citados foram: educação permanente em saúde, organização do serviço, comunicação, coordenação do cuidado e processo de enfermagem. A diversidade de indicadores demonstra falta de uniformização desses dados.

Assim, este estudo contribui para a construção de indicadores de qualidade ambulatorial de enfermagem, na tentativa de atender às necessidades de aprofundamento na produção de conhecimento em nível ambulatorial. Os indicadores de qualidade fazem parte de uma estratégia para o mapeamento das dificuldades e das necessidades de investimento nas estruturas dos serviços de saúde, podendo ser utilizados para direcionar e amparar ações e decisões referentes a condutas assistenciais, garantindo cuidados de enfermagem confiáveis, seguros e eficazes. Na ausência de métodos apropriados para a avaliação dos indicadores, baixos índices podem, na realidade, indicar fragilidade e inadequações nos cuidados de saúde oferecidos, como subnotificações, falhas na detecção de agravos e tratamentos inadequados.

Garantir o acesso a serviços de saúde de qualidade é fundamental. Para isso, é necessário que os diferentes níveis de atenção à saúde trabalhem em conjunto, evitando a sobrecarga dos serviços de saúde e a precarização da assistência, o que leva ao adoecimento dos profissionais, reinternações consecutivas e a cronificação de processos agudos.

# Translation: Calidad de la atención de enfermería ambulatoria: revisión de alcance*

**DOI:** 10.1590/1518-8345.7028.4523

**Authors:** Bruna Noschang de Brum, Carlise Rigon Dalla Nora, Adriana Roese Ramos, Luciana Foppa, Deise Lisboa Riquinho

**Affiliations:** 1 Universidade Federal do Rio Grande do Sul, Escola de Enfermagem e de Saúde Coletiva, Porto Alegre, RS, Brasil.; 2 Becaria de la Coordenação de Aperfeiçoamento de Pessoal de Nível Superior (CAPES), Brasil.; 3 Hospital de Clínicas de Porto Alegre, Serviço de Enfermagem Ambulatorial, Porto Alegre, RS, Brasil.

**Keywords:** Atención Ambulatoria, Atención Secundaria de Salud, Enfermería, Atención de Enfermería, Indicadores de Calidad de la Atención de Salud, Calidad de la Atención de Salud

## Abstract

mapear las producciones científicas nacionales e internacionales en salud sobre la calidad de la atención de enfermería ambulatoria.

revisión de alcance orientada por la Colaboración del *Joanna Briggs Institute*, realizada en 12 bases de datos y repositorios. Sólo se incluyeron artículos originales sobre la evaluación de la calidad de la atención de enfermería ambulatoria. No hubo delimitación de tiempo, metodología o idioma. Los datos fueron mapeados y organizados a través de la descripción temática y estadística de los artículos. Esta revisión fue registrada en la plataforma *Open Science Framework*.

se encontraron 45 estudios publicados entre 1984 y 2021, que dieron origen a 17 indicadores de calidad de la atención de enfermería ambulatoria que fueron agrupados según la tríada de Donabedian: cuatro de estructura, siete de proceso y seis de resultados. El área de atención predominante fue la oncología. El 55,3% de los estudios analizados se centró en los pacientes. Los indicadores más citados en los estudios fueron: educación continua en salud, organización del servicio, comunicación, coordinación de la atención y proceso de enfermería.

el pequeño número de estudios que hay en este campo, en comparación con otras áreas de la enfermería, demuestra lo poco que se estudia el tema a nivel nacional e internacional. Además, la diversidad de indicadores encontrados indica falta de estandarización de los datos.

## Destacados

**(1)** Pocas investigaciones abordan la evaluación de la calidad del servicio ambulatorio de enfermería. **(2)** Los indicadores de calidad mapean las dificultades en los servicios de salud. **(3)** Las publicaciones sobre calidad del servicio ambulatorio de enfermería han aumentado desde el año 2000. **(4)** La mayor parte de las investigaciones evaluadas se centraban en pacientes ambulatorios. **(5)** El indicador de calidad más citado en los estudios fue la Educación Continua en Salud.

## Introducción

La atención ambulatoria se refiere a la atención de salud que se brinda en ese nivel de atención, y se caracteriza fundamentalmente por la no hospitalización de los pacientes. Dichos escenarios de atención se están ampliando debido a cambios en el estándar de atención de salud, al envejecimiento poblacional y al aumento de la prevalencia de enfermedades crónicas^(1-3)^. Por ello, se busca mejorar la calidad del acceso a la atención de salud, con el objetivo de orientar la toma de decisiones^(4)^, a fin de adaptarlas para promover mejoras en la atención.

La evaluación de la calidad de la atención en salud, ampliamente utilizada como forma de categorizar indicadores, busca contemplar diferentes perspectivas tanto de los usuarios y profesionales, como de los proveedores de recursos de diferentes órdenes^(5)^. Se basa en la tríada de dimensiones de Donabedian: estructura, procesos y resultados. La dimensión estructura abarca los recursos humanos, físicos, materiales y financieros necesarios para brindar atención^(5)^. La dimensión proceso comprende las actividades que realizan los profesionales de la salud y los usuarios, como diagnóstico, tratamiento, relación usuario-equipo^(5)^. La dimensión resultados, a su vez, corresponde al producto final de la asistencia, y considera la salud, la satisfacción y las expectativas de los usuarios y profesionales^(5)^. Esta categorización permite realizar inferencias sobre los resultados de la atención, así como sobre los procesos que los preceden o el ambiente en el que ocurren^(5)^.

Se destacan dos estudios disponibles en la literatura sobre indicadores de calidad de los servicios ambulatorios sensibles a enfermería^(6-7)^. La revisión de la literatura destacó los siguientes indicadores en un estudio *:* cambio en la gravedad de los síntomas (*change in symptom severity*); fuerza de la alianza terapéutica (*strength of the therapeutic aliance*); uso de servicios de salud (*utilization of services*); satisfacción del paciente (*client satisfaction*); reducción de riesgos (*risk reduction*); aumento de los factores protectores (*increase in protective factors*) y estado funcional (*level of function/functional status*)^(6)^. Y en el otro, los indicadores mencionados fueron: conciliación de medicamentos (*medication reconciliation*); control de la hipertensión (*controlling high blood pressure*); evaluación de la depresión (*depression assessment conducted*); evaluación y seguimiento del dolor (*pain assessment and follow-up*) y reingreso del paciente (*hospital re-admissions*)^(7)^. Ambos enfatizan que es necesario que se lleven a cabo discusiones más profundas sobre los indicadores de calidad debido a la complejidad de las dimensiones que los componen, que dificultan determinar y aplicar métricas de calidad. Cabe señalar que los indicadores propuestos no incluyeron otras dimensiones importantes relacionadas con la atención ambulatoria. Sin embargo, el uso de indicadores de calidad en el área de enfermería es relevante, dado que permite visualizar las contribuciones que la atención de enfermería realiza a los resultados de los pacientes.

Durante las búsquedas se encontró sólo una revisión de alcance sobre el tema de enfermería ambulatoria^(8)^. Considerando los aspectos mencionados anteriormente, este estudio se centró en indicadores de evaluación de desempeño según el marco de Dubois y no incluyó investigaciones en español y portugués^(8)^, ello hace que la realización de esta revisión sea importante para producir indicadores de calidad que reflejen mejor la realidad brasileña y latinoamericana.

Dada la relevancia de la enfermería en la atención ambulatoria y considerando que preocuparse por el desarrollo de prácticas de atención de calidad en condiciones seguras forma parte de las funciones del enfermero, esta revisión tiene como objetivo mapear las producciones científicas nacionales e internacionales en salud sobre la calidad de la atención de enfermería ambulatoria.

## Método

### Protocolo y registro

Esta es una revisión de alcance, orientada por la Colaboración del *Joanna Briggs Institute* (JBI)^(9)^. Para garantizar la transparencia y la calidad, este estudio siguió las directrices presentes en el *Preferred Reporting Items for Systematic Reviews and Meta-Analyses extension for Scoping Reviews* (PRISMA-ScR)^(10)^. Esta revisión de alcance siguió las siguientes etapas: definir y adecuar el objetivo y la pregunta; desarrollar y adecuar los criterios de inclusión al objetivo y la pregunta; describir el enfoque; planificar la búsqueda de evidencia; selección, extracción de datos y presentación de la evidencia; búsqueda de la evidencia; selección de la evidencia; extracción de la evidencia; análisis de la evidencia; presentación de resultados; resumen de la evidencia en función del objetivo de la revisión, sacando conclusiones e implicancias de los hallazgos. El protocolo de la presente revisión fue registrado en la plataforma *Open Science Framework* (OSF)  bajo el *Digital Object Identifier* (DOI) https://doi.org/10.17605/OSF.IO/6YP7N.

### Criterios de elegibilidad

Los criterios de inclusión fueron artículos originales cuyos objetos incluyeran evaluar la calidad de la atención de enfermería ambulatoria, debido a la gran rigidez y robustez metodológica asociada a este tipo de estudio. Por ende, se incluyeron estudios con diseños cuantitativos, cualitativos o mixtos. Se enumeraron estudios cuyos participantes eran enfermeros involucrados en la atención ambulatoria en servicios de salud públicos o privados, pacientes atendidos por enfermeros en un ambiente ambulatorio especializado o estudios que describían actividades de atención de enfermería ambulatoria.

Los criterios de exclusión fueron: estudios teóricos, de revisión, metodológicos, de casos, editoriales, relatos de experiencia, disertaciones y tesis. No se establecieron límites de tiempo ni de idioma, con el objetivo de incluir el mayor número de estudios posible.

### Fuentes de información

Los estudios fueron seleccionados de diferentes repositorios y bases de datos: Literatura Latinoamericana y del Caribe en Ciencias de la Salud (LILACS), Índice Bibliográfico Español en Ciencias de la Salud (IBECS), *Medical Literature Analysis and Retrieval System Online* (Medline) y Base de Datos de Enfermería (BDENF) a través de la *Biblioteca Virtual em Saúde* (BVS); *Scientific Electronic Library Online* (SciELO); PubMed; *Web of Science Core Collection* a través de *Clarivate;* Embase y Scopus a través de Elsevier; *Cumulative Index to Nursing and Allied Health Literature* (CINAHL) a través de Ebsco. La búsqueda en la literatura gris se realizó mediante la herramienta *Google* Académico.

### Búsqueda en la literatura

La búsqueda de estudios se realizó el 22 de octubre de 2021 y se actualizó el 30 de abril de 2022.

Tanto la pregunta de investigación como la estrategia de búsqueda implementada en este estudio se desarrollaron con base en la mnemónica PCC, a saber: P: Población –es decir, publicaciones científicas nacionales e internacionales sobre la calidad de la atención de enfermería; C: Concepto –calidad de la atención de enfermería; y C: Contexto –servicios ambulatorios. Por ende, la pregunta orientadora elaborada fue: “¿qué dicen las publicaciones científicas nacionales e internacionales sobre la calidad de la atención de enfermería en los servicios ambulatorios?”.

La estrategia de búsqueda se definió considerando los Descriptores en Ciencias de la Salud (DeCS) y el *Medical Subject Headings* (MeSH) solo en inglés. Los descriptores seleccionados fueron: “atención ambulatoria”; “cuidados de enfermería”; “atención secundaria de salud”; “calidad de la atención en salud”, utilizando el operador booleano *AND*, respetando las particularidades y características de cada base de datos como se muestra en la Figura 1.

**Figura 1 -:** Estrategias de búsqueda en las bases de datos consultadas. Rio Grande do Sul, Brasil, 2023

**Bases de datos**	**Estrategia de búsqueda**
LILACS, IBECS, Medline y BDENF (a través de la *Biblioteca Virtual em Saúde* )	*(“Nursing Care”) AND (“Secondary Care”) AND (“Quality of Health Care”)* *(“Nursing Care”) AND (“Ambulatory Care”) AND (“Quality of Health Care”)*
CINAHL (a través de Ebsco)	*(“Nursing Care”) AND (“Secondary Care”) AND (“Quality of Health Care”)* *(“Nursing Care”) AND (“Ambulatory Care”) AND (“Quality of Health Care”)*
SciELO	*(Nursing Care) AND (Secondary Care) AND (Quality of Health Care)* *(Nursing Care) AND (Ambulatory Care) AND (Quality of Health Care)*
PubMed	*(Nursing Care) AND (Ambulatory Care) AND (Quality of Health Care)* *(“Nursing Care”) AND (“Ambulatory Care”)*
Embase (a través de *Elsevier* )	*nursing AND care AND ‘secondary health care’ AND ‘health care quality’* *nursing care’ AND ‘ambulatory care’ AND ‘health care quality’*
*Web of Science Core Collection* (a través de *Clarivate* )	*(Nursing Care) AND (Ambulatory Care) AND (Quality of Health Care)* *(Nursing Care) AND (Secondary Care) AND (Quality of Health Care)* *(Nursing Care) AND (Ambulatory Care)* *(“Nursing Care”) AND (“Secondary Care”)* *(“Ambulatory Care”) OR (“Secondary Care”) AND (“Nursing Care”)*
Scopus (a través de *Elsevier* )	*(“Nursing Care”) AND (“Ambulatory Care”) AND (“quality off health care”)* *(“Nursing Care”) AND (“Secondary Care”) AND (“quality off health care”)*
*Google* Académico	*“Nursing Care” AND “Ambulatory Care” AND “Quality of Health Care”*

### Selección de estudios

Para organizar los artículos, se almacenaron en el gestor de referencias *Zotero* ^©^. En cuanto a la selección de los estudios, los resultados de la búsqueda fueron analizados por dos investigadoras, de forma independiente, utilizando los formularios *Google Forms* ^©^ y *Google Sheets* ^©^. Los desacuerdos se resolvieron por consenso o con la presencia de un tercer investigador para la evaluación. Es decir, los investigadores compararon los resultados de las búsquedas realizadas, verificando si había diferencias en los hallazgos, intentando siempre incluir el mayor número de estudios posible.

### Extracción y análisis de datos

Para la etapa de extracción de datos se utilizó un formulario estructurado en la plataforma *Google Forms* ^©^ para identificar y describir los siguientes ítems: autor; año de publicación; país; revista; participantes; enfoque utilizado y principales resultados que se relacionaron con la calidad de la atención de enfermería ambulatoria, separándolos según las dimensiones estructura, procesos y resultados.

### Resumen de los resultados

Los datos se recolectaron de los resultados de los 45 estudios seleccionados, destacando la relación entre cada estudio y la calidad de la atención. La extracción de estos datos permitió mapearlos, interpretarlos y realizar un análisis numérico básico de la extensión, naturaleza y distribución de los estudios incluidos en la revisión. A continuación, se realizó la agrupación de los resultados por temática y descripción estadística en *Google Sheets* ^©^, con el objetivo de presentar una visión general de todo el material. Luego de organizar los resultados mediante la descripción temática, se agruparon en categorías según la tríada estructura, procesos y resultados propuesta por Donabedian^(5)^, utilizando cuadros en función de su relevancia.

El proceso de recolección de datos y selección de estudios se presentará en los resultados de este artículo a través de un diagrama de flujo que sigue las directrices PRISMA- ScR^(10)^.

## Resultados

Se identificaron 1.530 estudios a partir de bases de datos y repositorios. Después de identificar y excluir estudios duplicados, quedaron 794 estudios. Estos fueron evaluados mediante la lectura de los títulos y resúmenes, se excluyeron los que no abordaban la temática y se seleccionaron 225 estudios para lectura completa. Después de la lectura completa, se excluyeron 180 estudios: 119 no se ajustaban al tema de la revisión, 48 no cumplían los criterios de inclusión y 13 no se pudieron recuperar.

Los estudios fueron preseleccionados en base a la lectura de los títulos y resúmenes, y la muestra final se obtuvo mediante la lectura completa de los artículos, según el diagrama de flujo que se muestra en la Figura 2.

**Figura 2 -:** Diagrama de flujo de la selección de los estudios que componen la investigación adaptado de PRISMA ScR^(10)^. Rio Grande do Sul, Brasil, 2023

A partir de la selección de los artículos resultantes de las búsquedas en bases de datos y repositorios, se obtuvo una muestra compuesta por 45 artículos publicados entre 1984 y 2021. La ampliación del conocimiento en esta área de actuación se puede identificar por el aumento en el número de publicaciones a partir del año 2000, más concretamente de los años 2012 y 2018, dado que presentan un mayor número de estudios que el resto de los años.

Para facilitar la presentación, decidimos organizar los datos más relevantes de cada artículo en una tabla, como se puede ver en la Figura 3.

**Figura 3 -:** Caracterización de los artículos por autor, año de publicación, país en el que se realizó el estudio, idioma, enfoque, muestra y área de atención. Rio Grande do Sul, Brasil, 2023

**Cita**	**Año/País/Idioma**	**Enfoque**	**Muestra**	Área de Atención
Cunha, et al., 2021 ^(11)^	2021 Brasil Portugués	Cuantitativo (longitudinal)	Pacientes	Cardiología
Seibert, et al., 2020 ^(12)^	2020 Alemania Alemán	Cuantitativo (transversal)	Indicadores de calidad	Geriatría
Ameel, et al., 2020 ^(13)^	2020 Finlandia Inglés	Cualitativo (descriptivo)	Registros de enfermería*	Psiquiatría
Zendrato; Hariyati; Afifah, 2019 ^(14)^	2019 Indonesia Inglés	Cuantitativo (descriptivo)	Pacientes	Policlínico
Errico, et al., 2018 ^(15)^	2018 Brasil Inglés	Cuantitativo (transversal)	Registros de enfermería*	Obstetricia
Heale, et al., 2018 ^(16)^	2018 Canadá Inglés	Cuantitativo (transversal)	Registros de enfermería*	Endocrinología
Connor, et al., 2018 ^(17)^	2018 EE.UU. Inglés	Cuantitativo (descriptivo)	Enfermeros ^†^	Cardiología pediátrica
Seabra; Amendoeira; Sá, 2018 ^(18)^	2018 Portugal Inglés	Cuantitativo (transversal)	Pacientes | Psiquiatría ————————————–+———————————+ Profesionales de la salud Familiares de pacientes | Cardiología pediátrica	
Anderson, et al., 2018 ^(19)^	2018 EE.UU. Inglés	Cualitativo (descriptivo)		
Silva, et al., 2018 ^(20)^	2018 Brasil Inglés	Cualitativo (descriptivo)	Pacientes	Estomaterapia
Calvo; Sepulveda-Carrillo, 2017 ^(21)^	2017 Colombia Inglés	Cuantitativo (transversal)	Pacientes	Oncología
Ye, et al., 2016 ^(22)^	2016 EE.UU. Inglés	Cuantitativo (longitudinal)	Pacientes	Pediatría
Selvin, et al., 2016 ^(23)^	2016 Suiza Inglés	Cualitativo (descriptivo)	Pacientes	Psiquiatría
Macedo, et al., 2016 ^(24)^	2016 Brasil Inglés	Cualitativo (descriptivo)	Enfermeros ^†^	SAE VIH/SIDA
Vessey, et al., 2015 ^(25)^	2015 EE.UU. Inglés	Cualitativo (descriptivo)	Pacientes	Pediatría
Vanderboom; Thackeray; Rhudy, 2015 ^(26)^	2015 EE.UU. Inglés	Cualitativo (descriptivo)	Enfermeros ^†^	Policlínico
Tuna, et al., 2015 ^(27)^	2015 Turquía Inglés	Cuantitativo (descriptivo)	Enfermeros ^†^	Oncología
Komatsu; Yagasaki, 2014 ^(28)^	2014 Japón Inglés	Cualitativo (transversal)	Enfermeros ^†^	Oncología
Hammelef, et al., 2014 ^(29)^	2014 EE.UU. Inglés	Cuantitativo (transversal)	Pacientes	Oncología
Font Difour, et al., 2014 ^(30)^	2014 Cuba Español	Cuantitativo (descriptivo)	Pacientes y Enfermeros ^†^	Oncología
Armes, et al., 2014 ^(31)^	2014 Inglaterra Inglés	Cuantitativo (transversal)	Pacientes	Oncología
Van Den Bussche, et al., 2013 ^(32)^	2013 Alemania Alemán	Cuantitativo (descriptivo)	Profesionales de la salud	Psiquiatría
Palese, et al., 2013 ^(33)^	2013 Italia Inglés	Cuantitativo (transversal)	Registros de enfermería*	Oncología
Callen, et al., 2013 ^(34)^	2013 Australia Inglés	Mixto	Enfermeros ^†^	Reumatología
Williams, et al., 2012 ^(35)^	2012 Reino Unido Inglés	Cuantitativo (descriptivo)	Pacientes	Cardiología
Pinto, et al., 2012 ^(36)^	2012 Brasil Inglés	Cualitativo (descriptivo)	Enfermeros ^†^	Sin descripción
Pfeiffer, et al., 2012 ^(37)^	2012 EE.UU. Inglés	Mixto | Enfermeros ^†^		Sin descripción
Larsson, et al., 2012 ^(38)^	2012 Suecia Inglés	Cualitativo (descriptivo) | Pacientes		Reumatología
Kamimura, et al., 2012 ^(39)^	2012 EE.UU. Inglés	Cualitativo (descriptivo)	Enfermeros ^†^	Oncología
Friese; Manojlovich, 2012 ^(40)^	2012 EE.UU. Inglés	Mixto	Enfermeros ^†^	Oncología
Hjoerleifsdottir; Hallberg; Gunnarsdottir, 2010 ^(41)^	2010 Islandia Inglés	Cuantitativo (transversal)	Pacientes	Oncología
Skrutkowski, et al., 2008 ^(42)^	2008 Canadá Inglés	Cuantitativo (intervencionista)	Pacientes	Oncología
Rootmensen, et al., 2008 ^(43)^	2008 Países Bajos Inglés	Cuantitativo (intervencionista)	Pacientes	Neumología
Sisk, et al., 2006 ^(44)^	2006 EE.UU. Inglés	Cuantitativo (intervencionista)	Pacientes	Cardiología
Fonseca; Gutiérrez; Adami, 2006 ^(45)^	2006 Brasil Portugués	Cuantitativo (descriptivo)	Pacientes	Oncología
Mohrmann, et al., 2005 ^(46)^	2005 Alemania Alemán	Cuantitativo (descriptivo)	Pacientes	Sin descripción
Gesell; Gregory, 2004 ^(47)^	2004 EE.UU. Inglés	Cuantitativo (descriptivo)	Pacientes	Oncología
Cusack; Jones-Wells; Chisholm, 2004 ^(48)^	2004 EE.UU. Inglés	Cuantitativo (descriptivo)	Pacientes	Oncología
Arthur; Clifford, 2004 ^(49)^	2004 Reino Unido Inglés	Cuantitativo (transversal)	Pacientes	Reumatología
Zink, et al., 2000 ^(50)^	2000 Alemania Alemán	Cuantitativo (descriptivo)	Registros de enfermería*	Sin descripción
Oermann; Templin, 2000 ^(51)^	2000 EE.UU. Inglés	Cuantitativo (transversal)	Pacientes	Sin descripción
Oermann; Dillon; Templin, 2000 ^(52)^	2000 EE.UU. Inglés	Cuantitativo (descriptivo)	Pacientes	Sin descripción
Sanna, 1993 ^(53)^	1993 Brasil Portugués	Cualitativo (descriptivo)	Familiares de pacientes	Pediatría
Silva, 1985 ^(54)^	1985 Brasil Portugués	Cuantitativo (intervencionista	Pacientes	Endocrinología
Chang, et al., 1984 ^(55)^	1984 EE.UU. Inglés	Cuantitativo (intervencionista)	Pacientes	Geriatría

*Registros de enfermería = Incluye todo tipo de documentación completada por el equipo de enfermería; ^†^Enfermeros = Debido a la diversidad de lugares de estudio y a las diferentes organizaciones de la categoría enfermería, se decidió estandarizar la población como enfermeros, sin embargo, incluye a diferentes profesionales en esta categoría

Con el análisis de la Figura 3 se concluye que el 93,3% de los artículos fue publicado a partir del año 2000, el 80% de los artículos está en inglés^(13-29,31,33-44,47-49,51-52,55)^ y el 66,7% presenta un enfoque metodológico cuantitativo^(11-12,14-18,21-22,27,29-35,37,40-55)^. De los 19 lugares identificados, Estados Unidos^(17,19,22,25-26,29,37,39-40,44,47-48,51-52,55)^ fue el país con más artículos publicados, 33,3%, seguido del 12,8% en Brasil^(11,15,20,24,36,45,53-54)^ y del 8,9% en Alemania^(12,32,46,50)^.

En lo que respecta a la muestra del estudio, el 55,3% se centró en los pacientes^(11,14,18,20-23,25,29,31,35,38,41-49,51-52,54-55)^, seguido del 23,4 % cuyo foco fueron los enfermeros^(17,24,26-28,30,34,36-37,39-40)^, en el 10,6% fueron los registros de enfermería^(13,15-16,33,50)^, en el 4,3% los familiares de los pacientes^(19,53)^, en el 4,3% los profesionales de la salud^(19,32)^ y en el 2,1% los indicadores de calidad^(12)^. El área asistencial predominante en los artículos fue oncología^(21,27-31,33,39-42,45,47-48)^, con el 31,1% de las publicaciones.

Del agrupamiento de los artículos de esta revisión por temática, considerando la pregunta de investigación y el objetivo propuesto, surgieron 17 indicadores que inciden en la calidad de la atención de enfermería, y se presentan en la Figura 4. La distribución de estos indicadores se realizó de la siguiente forma: el 43,2% en la dimensión proceso^(12-23,25-28,30-35,37-39,41-54)^, el 30,3% en la dimensión resultado^(11,18,20-24,28-29,31-32,35-47,49,51-55)^ y el 26,5% en la dimensión estructura^(13-14,16-22,24,27,34-37,39-41,43-49,51-52,55)^.

**Figura 4 -:** Indicadores de calidad de la atención de enfermería ambulatoria según la tríada de estructura, procesos y resultados de Donabedian^(5)^. Rio Grande do Sul, Brasil, 2023

**Estructura**	**Proceso**	**Resultados**
Dimensionamiento del personal ^(27 , 36 , 39 - 40 , 45)^	Competencias de enfermería ^(30 - 31 , 34 - 35 , 37 , 40 , 46)^	Autocuidado ^(11 , 20 - 21 , 23 , 31 , 42 - 44 , 52 , 54)^
Educación continua en salud ^(14 , 16 , 19 , 24 , 36 - 37 , 43 , 45 - 46 , 48 - 49 , 51 - 52 , 55)^	Comunicación ^(14 , 16 , 22 - 23 , 32 , 37 - 41 , 51 - 52)^	Relación enfermero-equipo ^(24 , 32 , 37 , 39 - 40)^
Infraestructura ^(14 , 16 , 22 , 36 , 39 - 41 , 45 , 47)^	Coordinación de la atención ^(15 - 17 , 25 - 26 , 28 , 32 , 40 , 42 , 44 , 47 , 49)^	Relación enfermero-paciente ^(22 - 23 , 28 , 38 , 45 , 47 , 49 , 51 - 53 , 55)^
Organización del servicio ^(13 , 16 , 20 , 22 , 34 - 36 , 39 , 41 , 44 , 46 - 47 , 52)^	Cuidados de enfermería ^(13 , 16 , 18 , 20 , 27 , 41 , 43 , 46 , 48 , 53)^	Satisfacción del paciente ^(18 , 35 , 38 , 41 , 45 - 46 , 49 , 51 - 53 , 55)^
	Diagnóstico ^(12 , 15 , 18)^	Satisfacción profesional ^(36 - 37 , 39)^
	Educación para la salud ^(17 , 21 , 41 - 45 , 47 , 51 - 52 , 54)^	Salud mental ^(18 , 21 , 28 - 29 , 35 , 42 , 47)^
	Proceso de enfermería ^(13 - 16 , 19 , 26 , 30 , 31 , 46 - 48 , 50)^	

### Indicadores de calidad de la dimensión estructura

Los estudios incluidos en esta categoría fueron cuatro indicadores: dimensionamiento del personal, educación continua en salud, infraestructura y organización del servicio.

El dimensionamiento del personal^(27,36,39-40,45)^ incluye recursos humanos, carga de trabajo, dimensionamiento de los equipos de salud, número de pacientes por profesional según las necesidades asistenciales de cada paciente y el nivel de especialización requerido del profesional, además de la disponibilidad y accesibilidad de los profesionales de la salud.

La educación continua en salud^(14,16,19,24,36-37,43,45-46,48-49,51-52,55)^ se refiere a actividades educativas dirigidas a profesionales de la salud, e incluye capacidad técnica, conocimiento teórico y técnico, experiencia profesional, educación continua, retroalimentación y prácticas basadas en evidencia.

La infraestructura^(14,16,22,36,39-41,45,47)^ está relacionado con las instalaciones físicas de los servicios de salud, la disponibilidad de recursos materiales y comunitarios, el tiempo de espera para la atención y la accesibilidad de los servicios de salud.

La organización del servicio^(13,16,20,22,34-36,39,41,44,46-47,52)^ comprende aspectos organizativos de las entidades de salud, tales como acceso, equipo, modelo de atención vigente, actividades de gestión, procesos de auditoría y tecnologías de la información en salud.

### Indicadores de calidad de la dimensión proceso

Los estudios incluidos en esta categoría presentaron siete indicadores: competencias de enfermería, comunicación, coordinación de la atención, cuidados de enfermería, diagnóstico, educación para la salud y proceso de enfermería.

Las competencias de enfermería^(30-31,34-35,37,40,46)^ engloban actividades relacionadas con las competencias que deben tener los enfermeros como: liderazgo, gestión del equipo y de los recursos materiales, trabajo en equipo, manejo de conflictos, actividades administrativas, seguridad del paciente, supervisión y gestión de las actividades asistenciales.

La comunicación^(14,16,22-23,32,37-41,51-52)^ implica la capacidad del enfermero para transmitir conocimiento a los pacientes, familiares y la comunidad, y garantiza que los pacientes comprendan las indicaciones y que los profesionales entiendan las necesidades de los mismos en un intercambio de información. Esto requiere atención, empatía, sensibilidad, asertividad y respeto, eso facilita que la comunicación sea efectiva.

La coordinación de la atención^(15-17,25-26,28,32,40,42,44,47,49)^ se refiere a las actividades que se realizan en colaboración con otros profesionales de la salud, como derivación, continuidad de la atención según las necesidades del paciente, programación de consultas y exámenes, renovación de recetas, seguimiento del paciente y de la familia.

Los cuidados de enfermería^(13,16,18,20,27,41,43,46,48,53)^ implican actividades de atención directa al paciente, como intervenciones de enfermería, evaluación de la intensidad de los cuidados, características del equipo y de los pacientes, detección y atención integral. El diagnóstico^(12,15,18)^ se refiere a las patologías que presentan los pacientes según el Código Internacional de Enfermedades (CIE).

La educación para la salud^(17,21,41-45,47,51-52,54)^ abarca actividades educativas dirigidas a orientar a los pacientes y a la comunidad sobre los problemas de salud y la información para promover, prevenir y recuperar la salud*.* Los artículos mencionan el desarrollo de actividades de asesoramiento sobre hábitos saludables, información sobre enfermedades y procedimientos, prevención de eventos adversos y reducción del desperdicio de recursos materiales.

El proceso de enfermería^(13-16,19,26,30,31,46-48,50)^ se refiere a todas las actividades que involucran las cinco etapas del proceso de enfermería: recolección de datos, diagnóstico, planificación, implementación y evaluación, e incluye el registro de enfermería.

### Indicadores de calidad de la dimensión resultados

Los estudios incluidos en esta categoría presentaron seis indicadores: autocuidado, relación enfermero-equipo, relación enfermero-paciente, satisfacción del paciente, satisfacción profesional y salud mental.

El autocuidado^(11,20-21,23,31,42-44,52,54)^ está relacionado con la participación del paciente en el proceso de cuidado y en el manejo de signos y síntomas. La iniciativa del paciente y de los familiares para promover y mantener la salud, la adherencia al tratamiento y el desarrollo de actividades que alienten al paciente a convertirse en agente activo y corresponsable de su propio cuidado son parte de las estrategias citadas en los estudios.

La relación enfermero-equipo^(24,32,37,39-40)^ se refiere a la relación del enfermero con sus compañeros de trabajo, ya sean profesionales de enfermería u otros profesionales. Implica respeto, comprensión, confianza, comunicación, disponibilidad, comportamiento, autonomía y manejo de situaciones de conflicto.

La relación enfermero-paciente^(22-23,28,38,45,47,49,51-53,55)^ se relaciona con la dinámica del comportamiento entre enfermeros y pacientes. Estimular la autonomía, el seguimiento del paciente, los encuentros basados en la cordialidad, la empatía, la escucha activa, los sentimientos de comprensión, aceptación y sensibilidad, generan una sensación de confianza y seguridad.

La satisfacción del paciente^(18,35,38,41,45-46,49,51-53,55)^ se relaciona con la percepción del paciente sobre el resultado final de la atención. El nivel de satisfacción con la atención brindada, los valores, la participación, la conducta, la información relacionada con la salud, el vínculo con el profesional y el entorno influyen en la visión que tiene el paciente de la experiencia de la atención.

La satisfacción profesional^(36-37,39)^ se refiere a la percepción que tienen los profesionales de sus actividades laborales. El nivel de satisfacción o insatisfacción profesional está influenciado por las actividades que realiza, la dinámica del equipo de trabajo y las instalaciones de salud.

La salud mental^(18,21,28-29,35,42,47)^ abarca las necesidades emocionales de los pacientes y los profesionales. El grado de sufrimiento psicológico puede verse influenciado por los conflictos y el nivel de confianza entre los profesionales de la salud y los pacientes o entre los miembros del equipo de salud. El desarrollo de actividades educativas relacionadas con el bienestar mental y la derivación a apoyo emocional, según las necesidades de los individuos, puede reducir esos problemas.

## Discusión

Los resultados presentados en esta revisión ayudan a mapear la producción de conocimiento en el área de enfermería ambulatoria especializada. La mayoría de los estudios de esta revisión reflejan la realidad internacional de la práctica de enfermería, lo que puede dificultar aplicarlos en Brasil. La población de la mayoría de los artículos eran pacientes a los que les brindan atención los equipos de enfermería, lo que demuestra la importancia que tiene el paciente en el desarrollo de los niveles de calidad de los servicios^(11,14,18,20-23,25,29,31,35,38,41-49,51-52,54-55)^. Si bien ciertas especialidades de enfermería que actúan en atención ambulatoria tienen una mayor producción de estudios, como por ejemplo la oncología^(21,27-31,33,39-42,45,47-48)^, los mismos se desarrollaron en conjunto con el entorno hospitalario^(56)^, lo que dificulta determinar si los resultados reflejan correctamente la realidad ambulatoria.

La enfermería juega un papel importante en la atención ambulatoria, dado que implementa una atención centrada en el paciente y la familia. Por lo tanto, los indicadores de calidad deben reflejar la naturaleza de las intervenciones y sus preocupaciones^(8)^. En nuestros análisis, encontramos cuatro indicadores de salud relacionados con la estructura (dimensionamiento del personal, educación continua en salud, infraestructura, organización del servicio); siete indicadores de proceso (competencias de enfermería, comunicación, coordinación de la atención, cuidados de enfermería, diagnóstico, educación para la salud, proceso de enfermería); y seis de resultados (autocuidado, relación enfermero-equipo, relación enfermero-paciente, satisfacción del paciente, satisfacción profesional, salud mental). La literatura indica que el mayor foco está en el uso de indicadores de proceso para mejorar la calidad de los servicios, dado que tienen una fuerte asociación con los resultados^(56)^. Se observa que mejorar los indicadores en esas dos dimensiones puede tener impactos significativos en los servicios de salud^(56)^.

La conexión entre los cuidados de enfermería y el bienestar del paciente es compleja. No atender adecuadamente al personal de enfermería puede tener un profundo impacto en la calidad de la atención, lo que repercute negativamente en la salud de los pacientes^(8)^. Las cargas de trabajo excesivas, la falta de estructura organizacional en los servicios de salud y la rotación profesional comprometen el vínculo con la comunidad e influyen en la calidad de la atención^(56-57)^. Además, los cambios constantes de trabajadores provocan una sobrecarga en los equipos, dado que deben capacitar constantemente a los nuevos integrantes, lo que aumenta los costos y perjudica los procesos de trabajo^(57)^. Un número bajo de pacientes por enfermero se asocia con mejores resultados de salud^(8)^ y con lugares donde la proporción paciente-enfermero se basa en una atención de enfermería que realiza prácticas de atención de mejor calidad y basadas en evidencia^(8)^. En California, el *National Nurses United* utiliza un modelo de proporciones fijas de enfermeros por paciente y establece niveles obligatorios de profesionales que dependen del entorno y de las condiciones de salud de los pacientes^(58)^.

Las funciones de gestión del enfermero a veces se reducen a actividades de carácter burocrático y organizativo, llevando a una sobrecarga del profesional que debe gestionar la producción de la atención y el trabajo administrativo burocrático de gestión de personal y suministros^(57)^. A pesar de lo importante que es organizar el servicio de salud para evitar pérdidas en la continuación de la atención al paciente^(59-60)^, la burocratización y mecanización del trabajo de los enfermeros pone en riesgo la salud de los pacientes^(57)^, y puede llevar a que abandonen la atención directa y, en ocasiones, delegársela a los técnicos y auxiliares de enfermería^(57)^.

Algunos problemas de salud pueden requerir tratamiento especializado y coordinación entre los niveles de atención primaria y secundaria. La coordinación entre diferentes niveles de atención puede resultar difícil; la falta de confianza y conocimiento de los profesionales puede dar lugar a derivaciones innecesarias de pacientes a servicios especializados. Pero esos servicios suelen carecer de recursos suficientes y estar sobrecargados^(61)^. Las derivaciones son una parte importante del manejo de los pacientes, ya que garantizan la continuidad de la atención^(62)^. Un buen matriciamiento realizado por servicios especializados aumentaría la capacidad técnica y la confianza de los profesionales para atender esos casos, mejorar la resolución de la atención primaria y evitar derivaciones innecesarias^(61)^.

Algunos estudios informan que las dificultades para acceder a los servicios de salud son una de las principales razones del ausentismo en las consultas, lo que perjudica el seguimiento y la continuidad de la atención^(60)^. Se sugiere que, a medida que aumenta el porcentaje de continuidad, mejoran los resultados de salud^(56)^, ya que la continuidad promueve la creación de una relación de confianza entre el profesional y el paciente, que favorece la comunicación efectiva y mejora la adherencia al tratamiento^(56)^.

Los estudios que abordan la comunicación representaron una parte de nuestra muestra. Los pacientes identifican la comunicación como un indicador de atención en salud de alta calidad; una buena comunicación entre profesionales y pacientes genera mejores resultados^(63)^. Los atributos de comunicación con los pacientes deben incluir capacidad de escuchar, respeto, cortesía, explicaciones claras y lenguaje apropiado^(63)^. Además, también debe haber una comunicación adecuada entre profesionales y entre instituciones de salud^(63)^.

Algunos estudios abordan los registros de enfermería, que comprenden toda la documentación que completan los enfermeros y son esenciales para garantizar la calidad de la atención y la seguridad del paciente^(59)^. Documentar las etapas del proceso de enfermería –anamnesis y examen físico, diagnóstico de enfermería, prescripción de enfermería y evaluación^(64)^– es un instrumento importante para la continuidad de la atención, sirven como registro de las actividades realizadas por el equipo, garantizan el suministro de información importante sobre la asistencia y que se mantengan archivados datos relevantes en el proceso de auditoría^(59)^. Sin embargo, el registro de enfermería fue identificado como uno de los campos con mayor déficit de calidad en el proceso de enfermería en Brasil. Aunque se reconoce su importancia, la producción de registros adecuados enfrenta varias limitaciones en la práctica asistencial, como la escasez de profesionales, el exceso de trabajo y la falta de conocimiento teórico^(59)^.

Entre otras competencias que debe tener el enfermero se destacan la importancia del conocimiento técnico-científico, de la capacidad relacional y de la capacidad administrativa, asistencial y de gestión del personal. Mantener una relación adecuada con los pacientes, familiares y miembros del equipo de salud implica demostrar compromiso, participación y postura ética^(57)^. El liderazgo de enfermería es esencial para crear buenos ambientes laborales y tiene una fuerte conexión con la satisfacción profesional y la mejora de la salud mental. Sin embargo, su uso no se limita sólo a la gestión del equipo de salud, también es muy importante para promover los cuidados preventivos.

Reconocer que los pacientes son los mejores y los más calificados para brindar información sobre lo que es importante en la atención y las interacciones con los profesionales de la salud^(65)^ tiene un impacto importante en la calidad y la satisfacción del paciente, ya que la evidencia indica que hay asociación entre experiencia positiva, mejores resultados y mayor adherencia al tratamiento^(65)^. Los datos sobre la experiencia del paciente, que normalmente se obtienen a través de encuestas de satisfacción, se pueden utilizar como información sobre el desempeño en todos los niveles del sistema de salud, ya que proporcionan datos sólidos sobre la atención^(65-66)^.

El indicador educación para la salud influye en los resultados del paciente y es un componente clave de la práctica de enfermería^(56)^, dado que aumenta la capacidad de autogestión del paciente, lo que lleva a reducir los riesgos de empeoramiento de las condiciones de salud a corto y largo plazo^(56)^. Si bien la literatura menciona la importancia que tiene la educación para la salud para la calidad de la atención, pocos artículos detallan las actividades educativas implementadas o evalúan el impacto de dichas intervenciones en los pacientes.

La educación también es un factor importante en la formación de profesionales de la salud. La Organización Panamericana de la Salud (OPS) ya alertó sobre la formación de profesionales de la salud a través de un currículum universitario basado en paradigmas curativos, hospitalocéntrico y conocimiento fragmentado en salud, que valora las especialidades y deja de lado la concepción global del ser humano y de los procesos de salud y enfermedad^(57)^. Por lo tanto, la educación continua es una herramienta importante para la calificación del trabajo del enfermero y debe considerar cada contexto y las exigencias laborales^(57)^.

La práctica basada en la evidencia estuvo presente en los estudios analizados. Este método se define como un enfoque para resolver problemas de salud y mejorar la toma de decisiones, impulsado por la búsqueda de la mejor y más reciente evidencia, que incluye la experiencia clínica, la evaluación y las preferencias del paciente en un determinado contexto de salud. Los estudios han indicado que la práctica basada en evidencia aumenta la calidad de los sistemas de salud, mejora los resultados de los pacientes, reduce los costos y promueve una mayor satisfacción^(62)^. El indicador autocuidado está muy arraigado en el ámbito de la práctica de enfermería, dado que los enfermeros brindan habitualmente ese servicio a través de prácticas de educación para la salud^(67)^.

Los resultados de esta revisión contribuyen al avance del conocimiento científico en el área de enfermería en salud colectiva, especialmente para la calificación de la atención ambulatoria. La evidencia recopilada ayuda a llenar el vacío de conocimiento sobre los indicadores de calidad del servicio ambulatorio de enfermería, dado que los enfermeros desempeñan un papel importante en la gestión de la atención. Sin embargo, parece que hay barreras para traducir los resultados de la investigación en la práctica asistencial. Las futuras líneas de investigación en esta área pueden investigar cuáles son las barreras, ya que se los utiliza ampliamente en la atención hospitalaria desde hace mucho tiempo.

Se considera una limitación del estudio la dificultad para obtener investigaciones específicas en el área de enfermería ambulatoria especializada. Si bien el tema de la calidad del servicio ambulatorio de enfermería no es nuevo en la literatura, la producción de investigaciones en ese nivel de atención aún es pequeña en comparación con la gran cantidad de estudios producidos en el área hospitalaria y de atención primaria. Además, la dificultad para obtener artículos primarios centrados en la atención ambulatoria especializada limitó la posibilidad de hacer inferencias. Se necesitaría un conjunto más amplio de datos primarios para garantizar un análisis más profundo. Como resultado, las relaciones discutidas en este artículo se obtuvieron en su mayoría de estudios que se centraron principalmente en otros entornos ambulatorios.

El uso de diferentes descriptores o bases de indexación no incluidas en esta revisión podría permitir la obtención de otros estudios. Por otro lado, haber incluido solamente artículos originales, no haber utilizado descriptores en otros idiomas, como portugués y español, o sinónimos relevantes, así como omitir otros descriptores, como *Thesaurus* de CINAHL y *Emtree* de Embase, puede haber generado un sesgo en la identificación de los estudios. Por lo tanto, los autores reconocen que es posible que se hayan omitido importantes investigaciones publicadas utilizando nuestra estrategia de búsqueda.

Los estudios mapeados demuestran que hay desigualdad en las publicaciones nacionales e internacionales sobre el tema, poco explorado en la realidad brasileña, además de que existe una diversidad de indicadores que afectan la calidad de la atención de enfermería. Por ende, parece que hay un vacío en el conocimiento científico sobre la estandarización de los indicadores de calidad de la atención de enfermería ambulatoria.

## Conclusión

El mapeo de las producciones científicas nacionales e internacionales en salud sobre la calidad de la atención de enfermería ambulatoria demostró poca exploración del tema, especialmente cuando se compara con otras áreas del conocimiento en enfermería ambulatoria, tanto a nivel nacional como internacional. De la agrupación de los datos obtenidos del análisis por temas, surgieron 17 indicadores que inciden en la calidad de la atención de enfermería, la mayoría de los cuales se encuentran en la dimensión proceso. Los indicadores más citados fueron: educación continua en salud, organización del servicio, comunicación, coordinación de la atención y proceso de enfermería. La diversidad de indicadores demuestra que hay una falta de uniformidad en los datos.

Por lo tanto, este estudio contribuye a elaborar indicadores de calidad de enfermería ambulatoria, en el intento por satisfacer las necesidades de profundizar en la producción de conocimiento en el nivel ambulatorio. Los indicadores de calidad son parte de una estrategia para mapear las dificultades y las necesidades de invertir en las estructuras de los servicios de salud, y pueden ser utilizados para dirigir y apoyar acciones y decisiones relativas a las conductas asistenciales, que garantizan una atención de enfermería confiable, segura y efectiva. Ante la falta de métodos apropiados para evaluar los indicadores, los bajos índices pueden, en realidad, indicar debilidad e inadecuaciones en la atención de salud que se brinda, como subregistro, fallas en la detección de enfermedades y tratamientos inadecuados.

Es fundamental garantizar el acceso a servicios de salud de calidad. Para lograrlo, es necesario que los diferentes niveles de atención de salud trabajen juntos, evitando la sobrecarga de los servicios de salud y la precariedad de la atención, que conducen a la enfermedad de los profesionales, los reingresos consecutivos y la cronificación de procesos agudos.
